# Recent Advances in Metal–Organic Frameworks Derived Nanocomposites for Photocatalytic Applications in Energy and Environment

**DOI:** 10.1002/advs.202100625

**Published:** 2021-05-24

**Authors:** Mian Zahid Hussain, Zhuxian Yang, Zheng Huang, Quanli Jia, Yanqiu Zhu, Yongde Xia

**Affiliations:** ^1^ College of Engineering Mathematics and Physical Sciences University of Exeter Exeter EX4 4QF UK; ^2^ Henan Key Laboratory of High Temperature Functional Ceramics Zhengzhou University Zhengzhou 450052 China

**Keywords:** metal–organic framework derivatives, nanocomposites, photocatalysis, photocatalytic CO_2_ reduction, photocatalytic H_2_ evolution, photodegradation

## Abstract

Solar energy is a key sustainable energy resource, and materials with optimal properties are essential for efficient solar energy‐driven applications in photocatalysis. Metal–organic frameworks (MOFs) are excellent platforms to generate different nanocomposites comprising metals, oxides, chalcogenides, phosphides, or carbides embedded in porous carbon matrix. These MOF derived nanocomposites offer symbiosis of properties like high crystallinities, inherited morphologies, controllable dimensions, and tunable textural properties. Particularly, adjustable energy band positions achieved by in situ tailored self/external doping and controllable surface functionalities make these nanocomposites promising photocatalysts. Despite some progress in this field, fundamental questions remain to be addressed to further understand the relationship between the structures, properties, and photocatalytic performance of nanocomposites. In this review, different synthesis approaches including self‐template and external‐template methods to produce MOF derived nanocomposites with various dimensions (0D, 1D, 2D, or 3D), morphologies, chemical compositions, energy bandgaps, and surface functionalities are comprehensively summarized and analyzed. The state‐of‐the‐art progress in the applications of MOF derived nanocomposites in photocatalytic water splitting for H_2_ generation, photodegradation of organic pollutants, and photocatalytic CO_2_ reduction are systemically reviewed. The relationships between the nanocomposite properties and their photocatalytic performance are highlighted, and the perspectives of MOF derived nanocomposites for photocatalytic applications are also discussed.

## Graphical abstract

The state‐of‐the‐art progress in the production of metal–organic framework (MOF) derived nanocomposites with various dimensions (0D, 1D, 2D, or 3D), morphologies, chemical compositions, energy bandgaps, and surface functionalities are comprehensively summarized and analyzed. The photocatalytic applications of those MOF derived nanocomposites in photocatalytic water splitting for H_2_ generation, photodegradation of organic pollutants, and photocatalytic CO_2_ reduction are systemically reviewed.

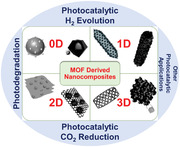

## 1 Introduction

The forthcoming depletion of fossil fuel‐based energy resources such as oils, gases, and coals makes it compelling to develop alternative clean and cost‐effective energy resources to fulfil the ever‐growing worldwide energy demand. Concurrently, the release of toxic organic pollutants such as textile dyes, pharmaceutical contaminants, and antibiotics into the water system as well as the emission of greenhouse gases (such as carbon dioxide and methane) into the atmosphere from industries pose great challenges to the environment, which have caused water pollution, incurable global warming and climate change. On the other hand, due to the abundant availability of sunlight, solar energy has become a key renewable and sustainable energy resource and photocatalysis has emerged as one of the most attractive approaches to practically solve environmental and energy problems. A variety of nanomaterials including metal nanoparticles (NPs), metal oxides, alloys, carbons and their composites have been explored as photocatalysts for hydrogen (H_2_) generation by water splitting and photodegradation of organic pollutants in the wastewater.^[^ ^1^, ^2^, ^3^, ^4^ ^]^ Moreover, to mitigate the environmental damages caused by carbon dioxide (CO_2_) emission, it is important to reduce the amount of CO_2_ released into the atmosphere.^[^ ^5^ ^]^ One plausible approach is to capture CO_2_ from the industrial exhaust and chemically reduce it to carbon monoxide (CO) or other value‐added reusable hydrocarbons such as methane (CH_4_), methanol (CH_3_OH), ethanol (C_2_H_5_OH) and many other chemicals.^[^ ^6^, ^7^ ^]^ However, it remains a great challenge to find the appropriate photocatalytic materials with optimal properties and excellent performance to meet the practical applications. Metal–organic frameworks (MOFs) are one of the most promising materials, which offer viable solutions to tackle those above mentioned energy and environmental problems.

MOFs are a class of crystalline porous solids, which consist of metal ions and organic linkers (ligands) bound together by the coordination bonds between metal ions/clusters and organic ligands (called secondly building units, SBUs).^[^ ^8^, ^9^ ^]^ When MOFs were first synthesized in the 1990s, they were initially studied mainly for gas separation and storage.^[^ ^9^, ^10^ ^]^ Later, MOFs were widely investigated for other applications such as sensing, energy storage and conversion, drug delivery, optoelectronics, batteries, supercapacitors, fuel cells and catalysis.^[^ ^5^, ^11^, ^12^, ^13^, ^14^, ^15^, ^16^, ^17^, ^18^, ^19^, ^20^, ^21^, ^22^, ^23^, ^24^, ^25^, ^26^ ^]^ Since the past few years, MOFs have further been used in solar energy‐driven applications especially as photocatalysts for H_2_ evolution (by water splitting), dye degradation and CO_2_ reduction.^[^ ^16^, ^18^, ^27^, ^28^, ^29^, ^30^, ^31^, ^32^, ^33^, ^34^, ^35^, ^36^ ^]^ The moderate strength of coordination bonds between the metal nodes and organic ligands, the wide energy bandgaps and the poor semiconducting properties (with limited electric charge generation and charge transfer) of pristine MOFs make them less favorable to be directly employed in photocatalytic applications.^[^ ^37^, ^38^, ^39^, ^40^, ^41^ ^]^ However, their distinct features such as rationally designed structures, high crystallinity, engineered morphologies, high specific surface area and tunable porosities make them exceptional platform materials as precursors or sacrificial templates to generate porous carbon (PC)‐based nanocomposites for various applications.^[^ ^14^, ^21^, ^23^, ^42^, ^43^, ^44^, ^45^, ^46^, ^47^, ^48^, ^49^, ^50^, ^51^, ^52^ ^]^

Many excellent reviews on the synthesis and applications of MOF derived metals, metal oxides, and porous carbons have been published in the past several years.^[^ ^5^, ^17^, ^18^, ^23^, ^25^, ^33^, ^36^, ^39^, ^40^, ^45^, ^46^, ^47^, ^53^, ^54^, ^55^, ^56^, ^57^, ^58^, ^59^, ^60^, ^61^ ^]^ However, to the best of our knowledge, so far no dedicated reports are available to provide critical analysis and overview on MOF derived nanocomposites for photocatalytic applications, which is pivotal for further design and development of advanced photocatalytic nanocomposites for highly efficient solar energy‐driven utilizations. In this work, we aim to overview the generation of MOF derived composite materials focusing on their applications in one of the most important renewable energy technologies—photocatalysis. Not only the state‐of‐the‐art progress in MOF derived nanocomposite materials for solar energy‐driven utilization are summarized, but also highlights on their structure–property–performance relationship are provided. In particular, a detailed review on the synthesis methods of MOF derived nanocomposites and the optimization strategies, which can enable better control over particle sizes, chemical compositions, morphologies, textural properties, in situ energy bandgap engineering, crystalline phase compositions and surface‐functionalization for improved photocatalytic performance is presented. In this review, we first briefly summarize the basic principles of the synthesis of MOF derived nanocomposites and discuss the photocatalysis mechanisms of nanomaterials in the typical applications including photocatalytic H_2_ evolution from water splitting, photodegradation of organic pollutants, and photocatalytic CO_2_ reduction. We then focus on the recent progress on the optimization of physicochemical properties and the applications of these MOF derived nanocomposite materials in various photocatalysis fields in detail based on their dimension (0D, 1D, 2D, and 3D) and morphology. Finally, we discuss the challenges and opportunities in further development of the structure–property–application relationship of MOF derived nanocomposites and offer some concluding remarks for future design of high‐performance photocatalytic nanocomposite materials for renewable energy applications. It may inspire ideas for future development of advanced photocatalytic materials for clean energy utilization and mitigation of environmental pollutants.

## 2 Basic Principles of the Synthesis of MOF Derived Nanocomposites

MOFs can be directly pyrolyzed to derive metal oxides, metal oxide/carbon composites and highly porous carbons under suitable conditions.^[^ ^24^, ^44^, ^45^, ^48^, ^62^ ^]^ The morphology of the derived materials depends upon the morphology of the i) initial MOFs used as precursors or sacrificial templates; ii) the pyrolysis temperature; iii) the gas atmosphere and iv) other parameters. The MOF precursors can be divided into two categories as self‐templated MOFs and external‐templated MOFs.^[^ ^39^ ^]^ The self‐templated MOFs include pristine MOF structures constructed by the self‐assembly of metal ions/clusters and organic linkers. They consist of single metal or multimetal, encapsulated metals and heteroatom (N, C, S, and P) doped metal(s) either directly loaded or modified through postsynthesis treatment, impregnation or ion/linker exchange. The morphology of these self‐templated MOFs stems solely from the reticular structure formed by the coordination bonds between metal clusters and organic linkers, which do not change after the introducing of guest species.^[^ ^11^, ^63^, ^64^ ^]^ These self‐templated MOFs can readily replicate their morphologies into the MOF derived nanocomposites. As for the external‐templated MOFs, they primarily consist of composite MOFs combined with other materials. They can be prepared by mechanical mixing or in situ growth of MOFs on a supporting external‐template such as spherical SiO_2_, layered‐like g‐C_3_N_4,_ graphene oxide (GO) or reduced graphene oxide (rGO). In this case, the morphology of the MOF derived nanocomposites mainly depends upon the morphology of the supporting external‐templates.^[^ ^32^, ^39^ ^]^ Generally, MOFs and their derived nanocomposites can be classified as 0D, 1D, 2D, and 3D (D stands for dimension). While those nanocomposites, which possess more or less spherical shapes such as polyhedra, core/shell and hollow structures are regarded as 0D materials; those composites with structural shapes like nanorods, nanoshuttles, nanotubes, or nanowires are considered as 1D materials; nanocomposites such as nanoplatelets and nanosheets with a thickness in the nanometer range are categorized as 2D materials, and those possessing the shapes/morphologies like nanodiscs, nanocubes, nanocages, and nanosponges are regarded as 3D materials.^[^ ^21^, ^39^, ^55^, ^65^ ^]^

Early reports suggested that MOFs decompose at high temperature and lose their morphologies to form featureless bulk materials.^[^ ^66^ ^]^ However, recent studies reveal that if rationally designed MOF precursors are selected, under controlled pyrolysis conditions, MOF derived nanomaterials tend to retain the morphologies of the MOF precursors.^[^ ^14^, ^67^ ^]^ By carefully choosing the suitable MOF precursors, a variety of 0D, 1D, 2D, and 3D nanomaterials that possess robust structural and chemical stabilities, desired morphologies, adjustable textural properties and various surface functionalities can be derived.^[^ ^11^, ^36^, ^53^, ^55^, ^68^, ^69^ ^]^ In addition, the crystallite sizes, morphologies, chemical compositions, atomic and weight percentages and optimized crystalline phases of the resultant nanocomposites can be readily controlled by tuning the pyrolysis conditions including temperature, dwell time, heating rate and gaseous atmosphere.^[^ ^44^, ^48^, ^70^ ^]^ Compared with the conventional approaches to prepare photocatalysts, the carbonization of MOF precursor method not only offers a simple way to control the derivatives, but also shows a great advantage of in situ modification of the textural, electronic and semiconducting properties of the resulting materials, which are crucial to obtain high performance photocatalysts.^[^ ^71^, ^72^, ^73^, ^74^ ^]^ For instance, by using the heteroatom‐containing pristine MOFs or MOFs introduced with metal/nonmetal guest species as precursors, the resulting nanocomposites can be in situ doped with metals and/or heteroatoms such as N, C, S, and P to modify the energy band positions, electronic structures and semiconducting properties to enable broader sunlight absorption and improved charge separation.^[^ ^75^, ^76^, ^77^, ^78^ ^]^ Interestingly, in MOF derived nanocomposites, the crystalline phases and atomic ratios of metal oxides (TiO*_x_*, FeO*_x_*, CuO*_x_*, etc.) embedded in the carbon matrix can be in situ adjusted by controlling the pyrolysis temperature and gas atmosphere to optimize for high efficient photocatalysis.^[^ ^79^, ^80^, ^81^, ^82^ ^]^ Moreover, the porous carbons derived from MOFs can also be in situ functionalized with hydrophilic and/or hydrophobic functional groups via altering the pyrolysis gas atmospheres.^[^ ^54^, ^83^, ^84^ ^]^ **Table** **1** summarizes the key parameters and basic principles of MOFs derivatives.

**Table 1:** Summary of MOF precursors, basic principles of MOF derived composites and some important in situ modifiable properties that affect the photocatalytic performance

Metal–organic frameworks (MOFs)	
Self‐templated precursors	External‐templated precursors
Single‐metal MOFs Multimetal MOFs (encapsulated guest metals, heteroatoms, doped metals)	Single‐metal MOFs composite Multimetal MOF composites (mixed with or grown on different supports)
MOF derived nanocomposites by carbonization method	
Self‐templated MOF derivatives: preserve morphology and dimensionality of parent MOFs External‐templated MOF derivatives: adopts the morphology of the supporting external template	

Important preparation parameters of carbonization/pyrolysis			
Temperature	Ramp rate	Pyrolysis time	Gaseous atmosphere
One‐step heating or multistep heating	One‐step or multistep ramp rate		Inert gas: N_2_, Ar Oxidizing: Air, O_2_, H_2_O, CO_2_ Reducing: NH_3_, H_2_, H_2_S

General types of derived composites							
Metal oxide/carbon	Multimetal oxide/carbon	Metal phosphide/carbon	Metal/carbon	Metal sulfide/carbon	Metal nitride/carbon	Mixed metal compounds/carbon	Other composites

In situ tunable properties
Crystallinity and chemical compositions Textural properties Electronic and semiconducting properties Surface functionalization Catalytic active site engineering

Normally, at pyrolysis temperature higher than 350 °C, the well‐ordered MOF crystalline structures tend to collapse and the metal ions or clusters will transform into metals or metal compounds while the organic linkers will convert to amorphous or partially graphitic porous carbon matrices.^[^ ^45^, ^85^ ^]^ To obtain the MOF derived nanocomposites with desired morphologies, chemical compositions and structural properties, the following two basic parameters should be considered: i) The reduction potential of metal ions in MOFs; Das et al. reported that the metal ions in MOFs with reduction potential of −0.27 V or higher (such as Co^2+^, Cu^2+^, Ni^2+^, etc.) formed pure metal nanoparticles upon pyrolysis in an inert atmosphere whereas those having a reduction potential lower than −0.27 V (such as Zn^2+^, Ti^4+^, Fe^3+^, Al^3+^, etc.) formed metal oxides by reacting with the oxygen present in organic linker under an inert atmosphere;^[^ ^86^ ^]^ ii) The Tamman temperature of the metal species; it controls the homogeneous distribution and particle sizes of nanoparticles in MOF derived nanocomposites. The Tamman temperature, which is approximately equal to half of the melting point of the metal itself, is the point at which the atoms and molecules attain adequate energies for their bulk diffusion and can easily agglomerate and sinter.^[^ ^38^ ^]^ When MOF structures decompose at a higher temperature, the metal species begin to collide and form the respective nanoparticles such as pure metals, metal oxides, metal sulfides, metal carbides, metal phosphides, and/or their multimetallic combinations. Meanwhile, the organic linkers transform into carbon functioning as a barrier to keep the metal species localized and prevent their bulk agglomerations, which results in a homogeneous distribution of nanoparticles throughout the porous carbon matrix. Usually, as the pyrolysis temperature increases above 600 °C, relatively more carbon species in the MOF precursors evaporate in the form of CO or CO_2_ and concurrently larger metal or metal compound nanoparticles are formed due to the high temperature sintering process.^[^ ^38^ ^]^ Therefore, MOF precursors (depending on the type of metals) and the pyrolysis temperature together with the gas atmosphere are the crucial parameters to control the particle sizes, the chemical composition and morphologies of MOF derived nanocomposites.

## 3 Photocatalysis Mechanism of Nanocomposites

The fundamental reaction mechanisms of photocatalytic processes for semiconducting nanomaterials have already been covered in reviews and book chapters.^[^ ^87^, ^88^, ^89^, ^90^, ^91^ ^]^ Therefore, we only briefly discuss the reaction mechanisms and steps of photocatalysis for MOF derived nanocomposites in photocatalytic water splitting for H_2_ evolution, photodegradation of environmental pollutants and photocatalytic reduction of CO_2_. Photocatalysis, a term originated from the combination of photochemistry and catalysis, suggests that a photosensitive redox reaction can be accelerated by the presence of a photoactive semiconductor catalyst.^[^ ^92^ ^]^ In general, heterogeneous photocatalysis can be realized via the following three basic steps: a) absorption of photons and generation of electron and hole pairs; b) charge separation and diffusion to the surface and c) reduction and oxidation (redox) of the chemical species. These processes are described below:

a) Absorption of photons from the sunlight and generation of electron and hole pairs (e^−^/h^+^). If the energy of photons of the incident light is greater than the energy bandgap between valence bands (VBs) and conduction bands (CB) of the semiconductor material, the electrons (e^−^) jump from the valence band to the conduction band generating holes (h^+^) in the valence bands. The absorption of light and the generation of electron/hole pairs (e^−^/h^+^) depend upon multiple factors including energy bandgaps, absorption coefficient, densities of states, reflectance/scattering, and the absorption depths of nanocomposites. It is well understood that semiconducting metal compounds encapsulated in porous carbon matrix are better light absorbers compared to the pristine semiconducting nanoparticles.^[^ ^93^, ^94^, ^95^ ^]^
b) Charge separation and diffusion to the surface of the catalyst. The successful charge diffusion/migration depends upon the charge/carrier mobility, diffusion coefficient, carrier lifetime, diffusion length, carrier concentration and the charge recombination kinetics.^[^ ^96^ ^]^ In this critical step, the photoexcited charge carriers (e^−^ and h^+^) can recombine either on the surface of the catalyst or within the catalyst (bulk or volume recombination) very quickly (within nanoseconds) to dissipate their energies in the form of heat. The surface and bulk charge recombination are influenced by the crystallinity, particle sizes, surface/bulk defects and crystal structures (phase types and ratios) of the photocatalyst. Usually, the lower the number of defects in the catalyst, the higher crystallinity of the material. The presence of cocatalysts and the formation of heterojunctions inhibit the recombination of the photoexcited charge carriers by spatially separating and prolonging the lifetime of photoexcited charge carriers.^[^ ^72^, ^87^, ^97^, ^98^, ^99^ ^]^
c) Reduction and oxidation (redox) of the chemical species by the photoexcited electrons and holes. The morphology of the photocatalysts, the surface area, the active sites, metal cocatalysts and the surface functionalities play a critical role in this step. Therefore, it is of vital importance to optimize the morphologies, compositions and the interfacial contacts of metal compounds and carbon matrix in MOF derived nanocomposites to enhance photocatalytic performance.^[^ ^20^, ^94^, ^100^, ^101^ ^]^

In photodegradation of pollutants, the photogenerated holes (h^+^) in the valence band and the formed oxidant species such as hydroxyl (•OH) and superoxide (•O_2_^−^) radicals transform the organic pollutants into benign mineral acids, H_2_O and CO_2_. It is important to scavenge the photogenerated electrons to avoid the surface/bulk recombination of e^−^ and h^+^ pairs. It is observed that in most photodegradation reactions, superoxide radicals (•O_2_^−^) also produce hydroperoxyl radicals (HOO•) by further protonation which acts as electron scavengers.^[^ ^62^, ^91^ ^]^ The thermodynamic and kinetic studies via applying various models have been carried out to understand the adsorption and photocatalytic decomposition mechanisms of the organic pollutants. An important parameter in photocatalytic degradation of organic pollutant is the matching of energy levels (*E*_HOMO_ and *E*_LUMO_) between pollutant and (*E*_VB_ and *E*_CB_) of photocatalyst. Here, HOMO and LUMO stand for the highest occupied molecular orbital and the lowest unoccupied molecular orbital, respectively, and VB and CB are valence band and conduction band, respectively. If the *E*_HOMO_ energy level of the pollutant is more positive with respect to the normal hydrogen electrode (NHE) and the *E*_VB_ of a photocatalyst is more negative with respect to NHE, it may lead to higher photocatalytic activities for the photodegradation of the catalysts.^[^ ^102^, ^103^ ^]^ In general, factors such as the wavelength (UV, UV–visible, or full spectrum) and the flux of incident light, temperature, pH value of the media in the reactor, degradation energy levels of the pollutants, concentration of photocatalyst and organic pollutant, reaction time and charge carrier dynamics are key parameters determining the photodegradation performance.^[^ ^104^, ^105^ ^]^ The concentration of an organic pollutant can be monitored by measuring the intensity of the absorption peak, and the photodegradation efficiency of a photocatalyst can be determined by the following Equation (1)

(1) $$\mathrm{Photodegradation}\;\mathrm{efficiency}\left(\%\right)=\frac{C_{o}-C}{C_{o}}\times 100$$

where *C*_o_ and *C* are the initial (before irradiation at *t* = 0) and final (after irradiation for some time) concentrations of the organic pollutant in solution, respectively.^[^ ^91^ ^]^

The photocatalytic water splitting to generate H_2_ is a typical uphill (endothermic) reaction that requires the Gibbs free energy (Δ*G* = 238 kJ mol^−1^) to overcome the energy barrier, which means that the energy of the incident photons should be higher than 1.23 eV (*λ* < 1000 nm) to realize the reaction. Regarding the photocatalyst for H_2_ evolution from water splitting, two key requirements should be fulfilled: i) the energy bandgap between the valence and conduction bands should be above 1.23 eV and below 3.26 eV and ii) the valence band position (VB maxima) should be more positive than the oxidation potential of O_2_/H_2_O (1.23 eV vs NHE at pH = 0) whereas the conduction band position (CB minima) should be more negative than the reduction potential of H^+^/H_2_ (0 eV vs NHE at pH = 0). The doping of heteroatoms such as metallic or nonmetallic species, the formation of the heterojunctions and the interfacial contacts between the semiconductors and the carbon matrix can cause the narrowing of energy bandgaps, which dominantly result in enhanced light absorption and better charge separation.^[^ ^14^, ^88^, ^89^, ^106^ ^]^ An ideal photocatalyst for H_2_ evolution from water splitting would be the one which absorbs the maximum amount of photons in the UV‐vis and IR range because the visible light region accounts for a larger proportion of the electromagnetic spectrum. The apparent quantum yield (AQY%) at a certain wavelength of light can be calculated by the following Equation (2)^[^ ^106^ ^]^

(2) $$\mathrm{AQ}Y_{\lambda }=\frac{2\times \mathrm{Number}\;\mathrm{of}\;\mathrm{evolved}\;H_{2}\;\mathrm{molecules}\;}{\mathrm{Total}\;\mathrm{number}\;\mathrm{of}\;\mathrm{incident}\;\mathrm{photons}}\;\times 100\%$$

The photocatalytic CO_2_ reduction and conversion into CO or other hydrocarbons are rather complicated multistep reactions. CO_2_ is a chemically stable molecule with no dipole moment and possesses a linear geometry and closed‐shell configuration.^[^ ^107^ ^]^ However, the carbon atom in CO_2_ molecule can gain electrons which causes a repulsive force among the lone pair of electrons in oxygen atoms. It lowers the bond angle of O–C–O and activates the CO_2_ molecule to form carbonate‐like anion radical (CO_2_•^−^) species. The one electron‐based photoreduction is quite unlikely due to the very strong electrochemical potential (*E* ^o^_redox_ = – 1.90 V vs NHE) and practically no semiconducting nanomaterial offers such negative electrochemical potential. In the presence of multiple electrons and H_2_O (at pH = 7) as a proton donor, the CO_2_ molecules can be reduced to different hydrocarbon species by different no. of electrons and corresponding protons, such as formic acid (2e^−^), carbon monoxide (2e^−^), formaldehyde (4e^−^), methanol (6e^−^) and methane (8e^−^), respectively.^[^ ^87^, ^107^ ^]^ The possible photocatalytic reactions for CO_2_ reduction and conversion with corresponding electrochemical redox potential (vs NHE) are given below^[^ ^107^ ^]^

(3) $$CO_{2}+2H^{+}+2e^{-}\to \mathrm{HOOH}\;\;E_{\mathrm{redox}}^{o}\;=-0.61\;V$$

(4) $$CO_{2}+2H^{+}+2e^{-}\to \mathrm{CO}+H_{2}O\;\;E_{\mathrm{redox}}^{o}=-0.53\;V$$

(5) $$CO_{2}+4H^{+}+4e^{-}\to \mathrm{HCHO}+H_{2}O\;\;E_{\mathrm{redox}}^{o}=-0.48\;V$$

(6) $$CO_{2}+6H^{+}+6e^{-}\to CH_{3}\mathrm{OH}+H_{2}O\;\;E_{\mathrm{redox}}^{o}=-0.38\;V$$

(7) $$CO_{2}+8H^{+}+8e^{-}\to CH_{4}+2H_{2}O\;\;E_{\mathrm{redox}}^{o}=-0.24\;V$$

Similar to the photocatalytic H_2_ evolution from water splitting, the photocatalytic CO_2_ reduction also depends on many factors such as wavelength and intensity of the irradiated light, absorption efficiency, types of heterojunctions, energy band positions of the semiconductors, crystallinities, morphologies, specific surface area and other textural properties of the photocatalysts.^[^ ^5^, ^94^, ^107^, ^108^, ^109^ ^]^

## 4 MOF Derived Nanocomposites for Photocatalytic Applications

A variety of strategically selected MOFs with desired morphologies and tunable pore sizes can be transformed into nanocomposites by controlled thermal treatments.^[^ ^38^, ^47^ ^]^ The pyrolysis of thermally stable MOF precursors under inert gas atmosphere generates metals, metal oxides, metal sulfides, and metal phosphides embedded in porous carbon matrix with micro/mesoporous structures.^[^ ^12^ ^]^ Depending upon the pyrolysis conditions such as temperature, time, heating rate and gas atmosphere, these resulting nanocomposites can preserve the inherited morphologies of MOF precursors. The rational design of MOF precursors allows the derived nanocomposites in 0D, 1D, 2D, and 3D nanostructures with morphologies including polyhedral, core–shell structures, nanorods, nanotubes, nanocubes, nanodiscs and nanospheres.^[^ ^110^, ^111^, ^112^ ^]^ Moreover, the external‐templated MOF precursors can be transformed into hybrid composites such as carbon nanosheets (graphene, graphene oxide or carbon nitride) decorated with oxides, sulfides or phosphides in customizable dimensions.^[^ ^39^, ^113^ ^]^ In the past decade, MOF derived nanocomposites have shown great promise as highly efficient photocatalysts in some typical solar energy‐driven applications, outperforming the commercially available counterparts. This part of the review summarizes the reported strategies on morphology control and the physicochemical property tuning of the MOF derived nanocomposites, which optimize the performance in photocatalytic applications including photocatalytic water splitting for H_2_ evolution, photodegradation of organic pollutants and photocatalytic CO_2_ reduction.

**Figure 1:**
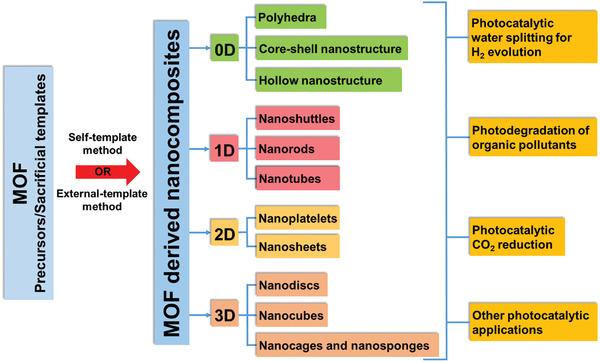
A schematic summary of the dimension and morphology of the MOF derived nanocomposites for photocatalytic applications.

The reported dimensions and morphologies of the MOF derived nanocomposites for various photocatalytic applications are summarized in **Figure** **1**. It is important to highlight that in this review 0D nanocomposites are not classified by the size and dimension of the nanoparticles because in MOF derived composites, each crystallite can consist of a large number of small nanoparticles. Therefore, the dimension and morphology of the MOFs crystallites and derived composites are classified in generic terms.^[^ ^39^, ^55^ ^]^

### 4.1 MOF Derived Nanocomposites in Photocatalytic Water Splitting for Hydrogen Evolution

The depletion of fossil fuels, environmental pollution and growing energy demand have made the H_2_ generated by photocatalytic water splitting to be one of the clean and cheap alternative energy sources. Since Fujishima and Honda discovered the possibility of mimicking photosynthesis to generate H_2_ using sunlight and water in 1972, tremendous efforts have been devoted to synthesizing highly efficient and stable heterogeneous catalysts for photocatalytic H_2_ evolution.^[^ ^92^ ^]^ It has been established that the photocatalytic H_2_ evolution strongly depends on the photoabsorption capability of the semiconducting catalysts, the accessibility of active sites, the energy bandgap positions and the charge separation/transfer efficiencies. The conventional semiconducting photocatalysts generally suffer from the low surface area, limited visible light absorption, inaccessible active sites and large energy bandgaps.^[^ ^48^ ^]^ Physicists and chemists have been trying to enhance the surface area by producing carbon‐based composite materials and optimize the energy bandgaps by introducing metal and/or nonmetal dopants and/or forming heterojunctions.^[^ ^88^, ^89^, ^90^, ^91^ ^]^ However, the maximization of visible light absorption for photocatalytic reactions is still the main challenge. New transition metal‐based photocatalysts (other than TiO_2_ and ZnO) such as oxides, sulfides and carbides have been demonstrated to obtain narrow energy bandgaps by introducing anionic and cationic dopants.^[^ ^88^, ^89^, ^114^, ^115^, ^116^ ^]^ MOF derived nanocomposites have exhibited a great potential to be used as photocatalysts for H_2_ evolution from water splitting since they offer symbiotic effects of tunable morphologies and chemical properties to enhance the photocatalytic performance.^[^ ^36^, ^38^, ^117^ ^]^ In the following section, MOF derived nanocomposites with different dimensions (0D, 1D, 2D, or 3D) for photocatalytic H_2_ production from water splitting is summarized. Moreover, the structure–morphology–application relationship as well as the physicochemical properties of MOF derived nanocomposites are analyzed and overviewed.

#### 4.1.1 0D Nanocomposites

##### Polyhedra

Among the self‐templated MOF derived 0D nanocomposites, the polyhedral morphologies are the earliest reported and the most common nanostructures with relatively high surface area and hierarchical pores.^[^ ^31^, ^75^, ^77^ ^]^ Transition metal‐based MOFs (such as Cu and Fe) can be employed to derive respective CuO*_x_* and Fe*_x_* O*_y_* nanoparticles due to their narrow energy bandgaps. For instance, Fe‐MOF derived octahedral Fe_2_O_3_/C nanocomposites show a reasonably good hydrogen evolution reaction (HER) performance under visible light. Xu et al. have reported that structurally and chemically stable composites obtained from self‐templated MOF precursor via a mild in situ carbonization process are highly efficient for H_2_ evolution under the white LED light. MIL‐101 (Fe) with ≈500 nm octahedral morphology transforms into magnetic metal oxide/carbon/oxyhydroxide (FeO_3.3_C_0.2_H_1.0_) nanocomposite that possesses the partial characteristics of *γ*‐Fe_2_O_3_, *α*‐FeOOH, and amorphous carbon. This octahedral shaped nanocomposite offers well‐exposed active sites with a specific surface area (Brunauer–Emmett–Teller (BET) ) of 93.7 m^2^ g^−1^ and wide pore diameter distribution in the range of 2–140 nm. In the presence of Eosin Y (EY), triethylamine in acetonitrile/water solution, this self‐templated MOF derived magnetic nanocomposite shows highly efficient H_2_ evolution activity of 4.2 mmol g_cat_^−1^ h^−1^ under the white LED light (400–700 nm) due to the synergistic effect of ferric oxide and O‐containing functional groups (such as —OH, —COOH) tethered on the surface of amorphous carbon. However, further investigations are required to understand the decomposition and transformation mechanism of the MOF precursors into the in situ formed products.^[^ ^82^ ^]^

Several studies have revealed that the charge transfer problems in MOF derived nanocomposites can be solved by the incorporation of conductive graphene or carbon‐based materials in semiconducting photocatalysts to form heterojunctions.^[^ ^95^, ^118^ ^]^ The hybrid nanocomposites fabricated by the combination of various counterparts (such as MOF derived metal oxides) and functionalized semiconducting carbon‐based materials with a narrow energy bandgap can further improve the charge separation and transfer due to the formation of heterojunction. For instance, Cu_2_O/C_3_N nanocomposites with octahedral morphology can be obtained by carbonizing self‐templated Cu‐MOF, HKUST‐1 loaded with urea (H_2_N)_2_CO as an additional nitrogen precursor at moderate temperature (above 450 °C) under nitrogen atmosphere. The p‐type Cu_2_O nanoparticles and nitrogen‐rich n‐type graphitic carbon form a p–n heterojunction resulting in an overall energy bandgap of 1.97 eV for the Cu_2_O/C_3_N composite. The p–n heterojunction facilitates the spatial separation and efficient transfer of photoinduced electrons from the Cu_2_O to the nitrogen‐rich carbon due to the interfacial contact, minimizing the charge recombination. This MOF derived Cu_2_O/C_3_N nanocomposite shows relatively higher H_2_ evolution activity under visible light compared to the pristine Cu_2_O and/or g‐C_3_N_4_, although its photocatalytic performance is poor compared to other reported MOF derived nanomaterials.^[^ ^119^ ^]^

Single‐metal oxides derived from MOFs, in general, show relatively poor light absorption capacities and quick charge recombination, which result in limited photocatalytic performance. Much effort has been devoted to engineering the energy bandgaps of multimetallic MOF derived nanocomposites to enhance the visible light absorption for improved H_2_ evolution due to the synergistic effects.^[^ ^70^, ^72^, ^78^ ^]^ Self‐templated bimetallic MOF/TiO_2_ precursors can be carbonized to obtain the nanocomposites that exhibit the symbiotic of properties. For example, TiO_2_–NiCoMOF–S_powder_, a mixture containing amorphous TiO_2_ nanoparticles encapsulated in Ni–Co–ZIF‐67 mixed with sulfur powder, can produce crystalline TiO_2_ nanoparticles well‐dispersed onto the PC shell decorated with Co/NiS nanoparticles (TiO_2_@CoNiS–PC) by pyrolyzing at 600 °C in an argon atmosphere. This TiO_2_@NiCoS–PC nanocomposite inherits the polyhedral morphologies of the precursor and exhibits moderate BET surface area of 93.5 m^2^ g^−1^. Under UV–vis light irradiation, the photoexcited electrons in TiO_2_ nanoparticles transfer to NiCoS/PC shell due to their lower overpotential and the holes are consumed by the sacrificial agent (MeOH). The spatial separation of photogenerated electrons and holes in well‐dispersed TiO_2_ inhibits the surface charge recombination because the conductive PC matrix facilitates the fast charge transfer. The NiCoS nanoparticles decorated on the PC matrix act as cocatalyst to offer additional active sites, which further improves the charge separation and the utilization efficiency of UV–vis light.^[^ ^117^ ^]^ Following this MOF‐templated approach, a variety of high performing multimetal oxides uniformly embedded in a conductive porous carbon matrix can be synthesized with desired morphologies and favorable physicochemical properties.

##### Hollow Nanocomposites

**Figure 2:**
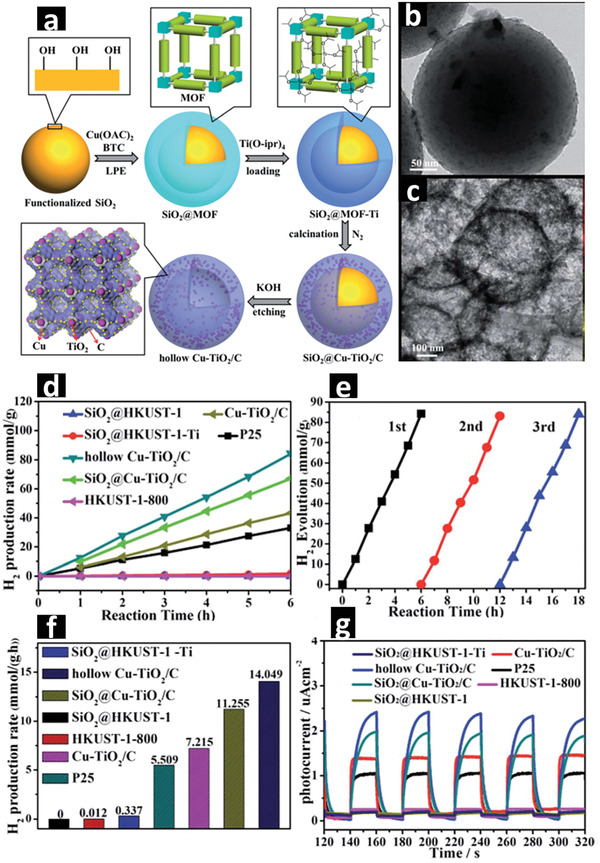
a) Schematic representation of hollow Cu–TiO_2_/C nanospheres derived from the external‐templated core–shell SiO_2_@SURMOF nanospheres. b,c) TEM images of SiO_2_@Cu–TiO_2_/C. d) Photocatalytic H_2_ evolution of MOF derived nanocomposites. e) The recyclability of best performing hollow Cu–TiO_2_/C nanospheres under solar irradiation. f) H_2_ evolution rates in mmol g_cat_^−1^ h^−1^ and g) photocurrent measurements of nanospheres under simulated solar irradiation. Adapted with permission.^[^ ^100^ ^]^ Copyright 2018, Royal Society of Chemistry.

Nanocomposites with 0D hollow spherical morphologies exhibit short path lengths and higher charge transfer efficiency, which could boost the photocatalytic H_2_ evolution. To date, no self‐templated MOF derived hollow nanostructures for photocatalytic H_2_ evolution have been reported, only a few external‐templated MOFs have been investigated to derive the hollow metal oxide/C composites by direct pyrolysis at high temperatures. For example, using the SiO_2_ sphere as an external template, a uniform thin shell of HKUST‐1 can be coated and grown on the surface of SiO_2_ spheres by a liquid phase epitaxial layer by layer immersion method. Then, a Ti‐containing precursor can be introduced to the SiO_2_@HKUST‐1 spheres, followed by a two‐step heat treatment at 400 and 800 °C under N_2_ atmosphere to generate the SiO_2_@Cu–TiO_2_/C composite. Finally, SiO_2_ in the composite can be etched in 1 m KOH aqueous solution at 80 °C for 12 h to obtain hollow Cu–TiO_2_/C nanospheres. The Cu_2_O and TiO_2_ nanoparticles homogeneously disperse in the porous carbon nanospheres and form p–n heterojunction at the interface that exhibits a bulk energy bandgap of 2.89 eV. As shown in **Figure** **2**, the Cu–TiO_2_/C nanocomposite with hollow spherical morphologies demonstrates a significant improvement of photocatalytic H_2_ evolution compared to the Cu–TiO_2_/C nanocomposite derived from HKUST‐1‐Ti prepared without using SiO_2_ as an external template. This twofold increase in photocatalytic H_2_ evolution performance under simulated sunlight is due to the improved light absorption, better separation and migration of photoexcited electrons/holes as well as the enhanced H_2_ diffusion.^[^ ^100^ ^]^ This method of using external‐templated MOF as precursors for the preparation of nanocomposite possessing the morphology of the external template can be extended to prepare other metal oxides/carbon nanospheres with single and/or multilayered complex morphologies for various photocatalytic and energy applications.

#### 4.1.2 1D Nanocomposites

Although recent reports have demonstrated that transition metal (e.g., Cd, Co, Ni) based MOF precursors (pristine or composite) can be used as sacrificial templates to obtain metal/multimetal oxides, sulfides, phosphides or carbides embedded in a 1D carbon matrix, MOF‐templated 1D nanocomposites are commonly derived from Fe‐MOFs. 1D MOF precursors with different morphologies such as nanorods, nanotubes and nanoshuttles can be synthesized by using specifically designed SBUs (metal ions/clusters), organic linkers and selected suitable solvents and controlling the ratios of chemical reactants.^[^ ^55^, ^120^, ^121^ ^]^ MOF derived 1D nanocomposites can be advantageous over bulk counterparts due to their enhanced surface with easily accessible active sites and controllable charge flow in a specific direction, which consequently shorten the charge migration/transfer distances in photocatalytic reactions. Though several studies on the synthesis and applications of 1D MOFs composites are available, only a few are dealing with photocatalytic water splitting for H_2_ evolution.^[^ ^13^, ^122^, ^123^ ^]^

##### Nanoshuttles

**Figure 3:**
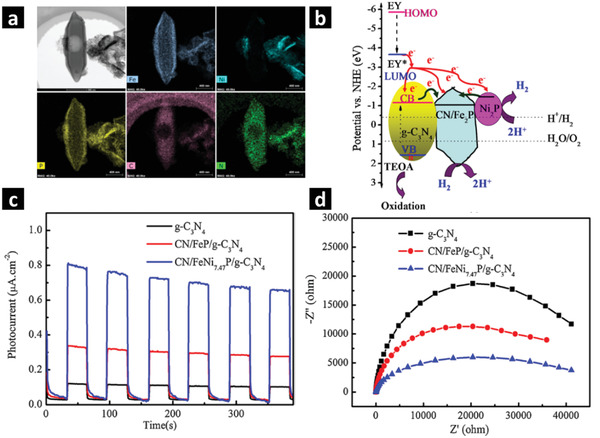
a) STEM‐EDX image and the elemental mapping of C–N/FeNi_7.47_P/g‐C_3_N_4_ composite for Fe, Ni, P, C, and N. b) Proposed mechanism of photocatalytic H_2_ evolution over the EY‐sensitized C–N/FeNiP/g‐C_3_N_4_ composite in the presence of sacrificial agent TEOA under irradiation of visible light. c) Transient photocurrent responses under visible light and d) EIS curves of external‐templated MOF derived optimized CN/FeNi_7.47_P/g‐C_3_N_4_ composites. Adapted with permission.^[^ ^76^ ^]^ Copyright 2019, Elsevier.

NH_2_‐MIL‐101(Fe), a MOF consisting of Fe oxo‐clusters and amino‐terephthalate, can be phosphidized to prepare FeP polyhedra with high BET surface area and exposed active sites. Moreover, if MOFs are anchored/grown on g‐C_3_N_4_, the FeP/g‐C_3_N_4_ composites with strong interfacial contact can be prepared for highly efficient photogenerated charge transfer. Taking advantage of these possibilities, Xu et al. used NH_2_‐MIL‐101(Fe) as a precursor to anchor the Ni(OH)_2_ nanoparticles.^[^ ^76^ ^]^ In the second step, g‐C_3_N_4_ nanosheets prepared by thermal treatment of melamine were deposited on the surface of as‐prepared NH_2_‐MIL‐101(Fe)/Ni(OH)_2_ composite under constant stirring at 60 °C overnight. The as‐prepared MIL‐101(Fe)/Ni(OH)_2_/g‐C_3_N_4_ sacrificial template with shuttle‐like morphology was then phosphidized in the presence of NaH_2_PO_2_ (with optimum mass ratios of 1:5) by heat treatment at 350 °C under N_2_ atmosphere, which resulted in C, N‐codoped FeNiP/g‐C_3_N_4_ nanocomposite. **Figure** **3** shows that the obtained 1D nanocomposite retains the inherited hexagonal microspindle (shuttle‐like) morphologies with all the elements homogeneously distributed throughout the sample. Under visible light irradiation, the optimized CN/FeNiP/g‐C_3_N_4_ nanocomposite with 1.0 mmol L^−1^ EY photosensitizer, exhibits exceptionally high H_2_ evolution activity of 13.81 mmol g_cat_^−1^ h^−1^ in 10% triethanolamine (TEOA) solution. The presence of EY sensitizer prolongs the lifetime of the photogenerated charge carriers compared to pure g‐C_3_N_4_. Furthermore, decorating the nanocomposite with Ni_2_P as a cocatalyst dramatically enhances the photocurrent response with the most efficient charge transfer. Without Ni_2_P, the plain CN/Fe_2_P/g‐C_3_N_4_ shows H_2_ evolution rate of 2.73 mmol g_cat_^−1^ h^−1^ under identical conditions whereas without adding EY photosensitizer, the CN/FeNiP/g‐C_3_N_4_ hexagonal nanoshuttles only exhibit H_2_ evolution rate of 0.196 mmol g_cat_^−1^ h^−1^. The optimized amount of EY photosensitizer and Ni_2_P play the crucial roles in this 1D CN/FeNiP/g‐C_3_N_4_ nanocomposite.^[^ ^76^ ^]^ The same strategy can be applied to fabricate a variety of complex multifunctionalized bimetal oxide/carbon composites via this method. However, it is worth noting that the involvement of multiple steps and a lack of simplicity make the nanocomposites obtained in this way less promising for practical applications. Simple one‐step methods are desirable to obtain scalable, highly efficient and stable photocatalysts for H_2_ evolution.

##### Nanorods

Theoretically, all MOFs can be pyrolyzed to derive composite nanomaterials. However, Fe‐MOF precursors have mostly been employed to yield 1D nanocomposites due to their 1D self‐assembly, water‐based synthesis and low toxicity.^[^ ^112^ ^]^ For example, self‐templated MOF, NH_2_‐MIL‐88B(Fe) can be directly carbonized at 600 °C in an inert atmosphere to fabricate magnetic 1D Fe_2_C nanorods without compromising the inherited morphology. The reduction potential of metal ions that constitute the MOF precursors plays a key role in determining the nature of metal species in the derived products. The in situ formed carbon (from the thermal decomposition of organic ligands in the MOF templates during the thermolysis process) is a strong reducing agent and the metal ions can be readily reduced to metal nanoparticles or form metal carbides at higher carbonization temperature in an inert atmosphere. The reduction potential and Gibbs free energies (Δ*G*) of the reactant species in MOF templates are vital in determining whether metals or metal carbide species can be formed.^[^ ^71^, ^86^ ^]^ Upon pyrolysis under Ar atmosphere, nanorod shaped NH_2_‐MIL‐88B(Fe) precursor with the rod length of ≈300 nm and width of ≈50 nm decomposes and transforms into crystalline Fe_2_C nanorods with shrunk sizes and coarse surfaces. These magnetic Fe_2_C nanorod shaped particles show relatively good performance in photocatalytic activity under visible light due to the well‐exposed active sites and easy charge transfer. The magnetic property makes these composites easy to collect after the reaction for recycling.^[^ ^124^ ^]^ Such a simple method can be optimized to fabricate desirable morphologies for other transition metal‐based MOFs. Furthermore, the formation mechanisms of porosities and the charge generation/charge transfer of the 1D MOF derivatives need to be deeply investigated by employing the in situ imaging and the operando spectroscopy techniques.

#### 4.1.3 2D Nanocomposites

It is of particular importance to synthesize 2D MOF sacrificial templates because of their promising physicochemical and optoelectrical properties. The 2D MOFs with desirable properties can be achieved by optimizing the compositions, growth of crystal phases, the formation of the surface/bulk defects and the textural properties for a variety of catalytic and energy applications.^[^ ^55^, ^125^ ^]^ Generally, self‐templated ultrathin 2D MOF precursors can be synthesized by various methods such as surfactant‐assisted bulk solutions (structure‐directing agent to direct the orientation of crystal growth), interfacial growth at air/liquid, liquid/liquid or liquid/solid interface, sonication/mechanical exfoliation and chemical intercalation.^[^ ^55^, ^125^, ^126^, ^127^, ^128^ ^]^ For external‐templated 2D MOF precursors, 2D carbon structures such as carbon nanosheets, graphene or graphene nitride are used, which have the benefit of combining the characteristics of MOFs and the external templates.^[^ ^129^, ^130^, ^131^ ^]^ However, developing direct synthesis methods to produce metal/multimetal 2D‐MOF precursors can be a convenient approach to overcome the complications of exfoliation or multistep external‐templating method.^[^ ^55^ ^]^ The controlled pyrolysis of 2D MOFs precursors can produce 2D nanosheets, nanofilms, and nanoflakes of porous carbon materials uniformly decorated with metal oxides, sulfides or phosphides, which can be excellent nanocomposites for photocatalytic H_2_ evolution applications.^[^ ^132^, ^133^ ^]^

##### Single‐Metallic Nanosheets

**Figure 4:**
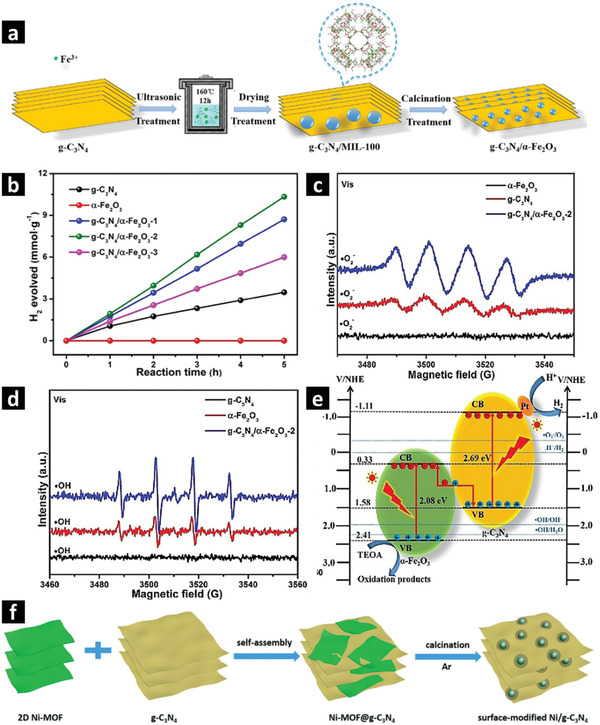
a) Schematic diagram shows the synthesis strategy of Fe‐MOF/g‐C_3_N_4_ derived g‐C_3_N_4_/*α*‐Fe_2_O_3_ nanocomposite. b) Photocatalytic H_2_ evolution by g‐C_3_N_4_/*α*‐Fe_2_O_3_ nanocomposite. c) EPR spectra measured in MeOH for DMPO‐•O_2_^−^. d) EPR spectra measured in aqueous solution for DMPO‐•OH. e) Photocatalytic H_2_ evolution mechanism of the g‐C_3_N_4_/*α*‐Fe_2_O_3_ nanocomposite under visible light (300 W Xe lamp and *λ* > 420 nm). a‐e) Adapted with permission.^[^ ^134^ ^]^ Copyright 2019, Wiley‐VCH. f) Schematic representation of 2D Ni‐MOF/g‐C_3_N_4_ derived Ni/g‐C_3_N_4_. Reproduced with permission.^[^ ^136^ ^]^ Copyright 2019, Royal Society of Chemistry.

Direct pyrolysis of hybrid MOF precursors (MOFs grown on 2D external templates) is a relatively simple method to fabricate MOF derived 2D metal oxide/C nanocomposites. Combining two semiconducting nanomaterials with finely tunable energy band positions can form heterojunctions that are desirable to enhance the photoexcited charge separation/transfer efficiencies. For example, Fe‐MOF derived *α*‐Fe_2_O_3_ nanoparticles with modified energy bandgaps coupled with carbon nitride (g‐C_3_N_4_) can form Z‐scheme heterojunctions through a rationally designed MOF‐template method.^[^ ^99^, ^131^, ^134^ ^]^ Melamine derived g‐C_3_N_4_ nanosheets can be used as external‐template to grow MIL‐100(Fe) on it, followed by calcination at 400 °C to obtain g‐C_3_N_4_/*α*‐Fe_2_O_3_ hybrid nanocomposite with 2D nanosheet morphology. As shown in **Figure** **4**a,b, MOF derived *α*‐Fe_2_O_3_ polyhedral nanoparticles uniformly dispersed on g‐C_3_N_4_ nanosheets with optimized composition and morphology exhibit a promising H_2_ evolution rate of 2.07 mmol g_cat_^−1^ h^−1^ under visible light in the presence of Pt cocatalyst. The charge generation/transfer mechanisms in the direct Z‐scheme heterojunctions can be investigated by electron paramagnetic resonance spectroscopy (EPR), transient photocurrent density measurement and electrochemical impedance spectroscopy (EIS) shown in Figure 4c–e. In traditional heterojunctions, photogenerated electrons and holes jump to the conduction band of g‐C_3_N_4_ and valence band of *α*‐Fe_2_O_3_, respectively. However, such charge mobility has not been observed in Z‐scheme heterojunction. Instead, the photogenerated electrons and holes in Z‐scheme g‐C_3_N_4_/*α*‐Fe_2_O_3_ nanocomposites stay spatially separated because the valence band position of g‐C_3_N_4_ (1.58 V vs NHE) is more negative than the standard redox potential of OH^−^/*OH (2.34 V vs NHE) and H_2_O/*OH (1.99 V vs NHE), while the conduction band position of *α*‐Fe_2_O_3_ (0.33 V vs NHE) is lower than that of the O_2_/*O_2_^−^ (−0.33 V vs NHE). Therefore, like traditional heterojunction, the optimized Z‐scheme heterojunctions such as g‐C_3_N_4_/*α*‐Fe_2_O_3_ can also enhance the visible light absorption, consequently accelerate the photogenerated charge separation and migration.^[^ ^134^ ^]^

Similarly, other transition metals (such as In, Ni) can also be loaded on g‐C_3_N_4_ nanosheets.^[^ ^135^, ^136^ ^]^ Surface modified Ni/g‐C_3_N_4_ nanosheets have been derived from external‐templated‐MOF composites.^[^ ^136^ ^]^ A 2D Ni‐MOF precursor was prepared by the ultrasonication method while g‐C_3_N_4_ nanosheets was derived by thermal polymerization of urea at 550 °C in air (Figure 4f). The as‐prepared precursors (2D Ni‐MOF and g‐C_3_N_4_) were mechanically mixed. The resulting mixture was pyrolyzed under argon atmosphere above 500 °C to obtain Ni/g‐C_3_N_4_ nanosheets, in which the 2D‐Ni‐MOF transforms into Ni and/or NiO forming Ni/NiO nanoparticles. The interfacial contacts between the Ni/NiO nanoparticles and the g‐C_3_N_4_ nanosheets offer enhanced light absorption as well as improved charge separation and transfer. Under visible light irradiation, the photogenerated electrons and holes carry out the redox reactions at the respective energy bands. The presence of Ni particles as a cocatalyst may contribute to the significant suppressing of the charge recombination. Adding EY as a photosensitizer, the optimized Ni/g‐C_3_N_4_ nanosheets exhibited high photocatalytic H_2_ evolution activity of 2.99 mmol g_cat_^−1^ h^−1^, attributed to the high conductivity of g‐C_3_N_4_ and efficient charge separation/transfer.^[^ ^136^ ^]^ A variety of transition metals such as Fe, Ni, Cu and Co‐based MOF derived metal/metal oxides can be studied as cocatalysts decorated on surface‐modified 2D nanosheets as an alternative to noble metals for effective H_2_ evolution from photocatalytic water splitting.

##### Multimetallic Nanosheets

Different from single metal or metal oxide‐based MOF derived composites, 2D nanocomposites with multimetallic oxide‐, sulfide‐ and phosphide‐based heterojunction can be excellent nanomaterials for photocatalytic applications due to their promising optoelectronic and photochemical properties.^[^ ^137^, ^138^, ^139^ ^]^ Metal sulfides such as CdS and ZnS with morphologies of nanosheets, nanoflowers and core/shell nanostructures have been extensively studied for photocatalytic applications. However, the low surface area, the environmental unfriendliness of CdS and the wide energy bandgap of ZnS together with their poor photostability limit their practical applications.^[^ ^15^, ^140^ ^]^ The challenges of poor photostability and the low surface area can be tackled by employing rationally designed bimetallic MOFs combined with external templates such as rGO or g‐C_3_N_4_ 2D nanosheets to fabricate stable metal and/or bimetal sulfide nanoparticles uniformly distributed in porous carbon matrix for enhanced photocatalytic H_2_ evolution. For example, ZnS/rGO/CuS nanocomposites can be derived from a mixture of ZIF‐8, thioacetamide (TAA), and rGO, which is obtained by simple solvothermal and ion‐exchange methods.^[^ ^132^ ^]^ The resulting ZnS/CuS nanoparticles retain the polyhedral morphology of ZIF‐8 precursor with particle size of ≈150 nm. These ZnS/CuS nanoparticles form heterojunctions and uniformly distribute in rGO 2D nanosheets, which enhances the photostability of ZnS/CuS nanoparticles and inhibit the agglomeration of the nanoparticles. The interfacial contacts between the ZnS/CuS nanoparticles and the conductive rGO nanosheets can facilitate the charge transfer of photoexcited electrons and holes, and consequently improve the spatial charge separation and transport efficiency. The external‐templated MOF derived porous ZnS/rGO/CuS 2D nanosheets with optimized 7 wt% Cu and 0.5 wt% rGO exhibit higher H_2_ evolution activity of 2.61 mmol g_cat_^−1^ h^−1^ under visible light compared to the counterpart bimetallic sulfide heterojunction derived from conventional inorganic precursors.^[^ ^132^ ^]^ This method may provide a viable route to synthesize noble metal‐free high efficient multimetallic sulfides/carbon nanocomposites for environment and energy applications.

The issues of bandgap and photostability of CdS nanoparticles can be addressed by adopting the external‐templated MOF method. Recently, Aleksandrzak et al. reported 2D nanosheets of Cd(OH)_2_/CdS/g‐C_3_N_4_/NPC nanocomposite derived from external‐templated MOF for H_2_ evolution under the solar simulator.^[^ ^141^ ^]^ Specifically, nanoporous carbon (NPC) matrix was obtained from the direct carbonization of Al‐MOF at 750 °C under inert atmosphere followed by washing with 17% HCl to remove the Al species. The g‐C_3_N_4_ nanosheets were derived from calcination of urea in a muffle furnace at 550 °C for 4 h under air. Finally, CdSO_4_ mixed with thiourea and NH_3_·H_2_O were added into g‐C_3_N_4_ and NPC under constant stirring followed by filtering and drying to collect Cd(OH)_2_/CdS/g‐C_3_N_4_/NPC nanocomposite. The resulting nanocomposite exhibited a BET surface area of 92.7 m^2^ g^−1^ and pore volume of 0.044 cm^3^ g^−1^. With the energy bandgap of 2.32 eV, 40 wt% of Cd(OH)_2_/CdS nanoparticles embedded in g‐C_3_N_4_/NPC matrix showed moderate H_2_ evolution activity of 148.13 µmol g_cat_^−1^ h^−1^ under the solar simulator. It is claimed that the formation of heterojunction between g‐C_3_N_4_ and Cd(OH)_2_/CdS nanoparticles can provide insights into the photoexcited charge generation and migration pathways. The photogenerated electrons in g‐C_3_N_4_ may migrate to Cd(OH)_2_/CdS compound due to its less negative conduction band maxima (CBM) compared to g‐C_3_N_4_. Then both the migrated electrons from g‐C_3_N_4_ and the electrons photogenerated in Cd(OH)_2_/CdS, may partially migrate to the NPC. A large number of catalytic active sites participate in water reduction due to the synergistic effects.^[^ ^141^ ^]^ Nonetheless, detailed in situ microscopic and photoelectrochemical characterizations are required to further understand the mechanism of heterojunction formation and charge transfer in this type of complex nanocomposites.

**Figure 5:**
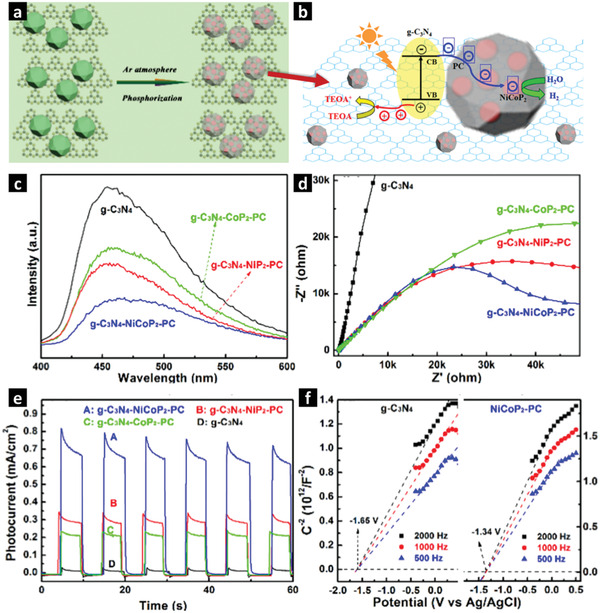
a) Schematic diagram of the g‐C_3_N_4_/MOF derived g‐C_3_N_4_–NiCoP_2_–PC nanocomposite. b) Proposed mechanism of photoinduced separation of charge carriers in the g‐C_3_N_4_–NiCoP_2_–PC composite and the reduction process for H_2_ evolution under UV–vis light. c) PL emission spectra, d) EIS spectra, e) transient photocurrent responses under irradiation of UV–vis light, and f) Mott–Schottky plots of g‐C_3_N_4_/NiCoP_2_–PC. Adapted with permission.^[^ ^133^ ^]^ Copyright 2019, American Chemical Society.

Similarly, bimetallic phosphide particles encapsulated in porous carbon hybrids and uniformly dispersed on 2D g‐C_3_N_4_ nanosheets have been derived by heat treatment of external‐templated bimetallic‐MOF precursors and the resulting composites are promising materials for photocatalytic applications. For example, as‐prepared bimetallic Ni–Co–C_6_H_3_(COOH)_3_, NaH_2_PO_2_, and g‐C_3_N_4_ were ground together in appropriate ratios, followed by phosphorization under an argon atmosphere at 350 °C for 2 h to obtain ternary g‐C_3_N_4_–Ni*_x_* Co_1−_ *_x_* P_2_–PC nanocomposite. The optimal ternary g‐C_3_N_4_–NiCoP_2_–PC nanocomposite (**Figure** **5**) with a BET surface area of 56.32 m^2^ g^−1^ showed a maximum H_2_ evolution activity of 2.1 mmol g_cat_^−1^ h^−1^ under UV–vis light in 20% TEOA/H_2_O solution, which was attributed to the low HER overpotential and the flat‐band potential of NiCoP_2_ as well as the high electrical conductivity of the MOF‐templated porous carbon/g‐C_3_N_4_.^[^ ^133^ ^]^ A library of MOF derived 2D multimetallic oxide‐, phosphide‐, and sulfide‐based nanocomposites with a variety of morphologies such as nanoplatelets, nanosheets and nanoribbons, can be designed and produced from selected bimetallic MOF precursors via self‐templated and external‐templated approaches. These new types of nanocomposites can effectively improve the photoexcited charge carrier transport, separation and utilization, which consequently enhances their photocatalytic H_2_ evolution performance. In **Table** **2**, a summary of the selected MOF derived nanocomposites for photocatalytic H_2_ evolution from water splitting is presented.

**Table 2:** Comparison of selected MOF derived nanocomposites for photocatalytic H_2_ evolution

			Experimental conditions			
MOF precursor	Catalyst	Morphology	Cocatalyst	Light source	H_2_ evolution [mmol g_cat_^−1^ h^−1^]	Refs.
HKUST‐1@urea	Cu_2_O@C_3_N	Octahedron	–	>420 nm 500 W Xe lamp	0.026	^[^ ^119^ ^]^
MIL‐101 (Fe)	Fe_2_O_3_/FeOOH/C	Octahedron	EY	>400 nm White LED	4.167	^[^ ^82^ ^]^
TiO_2_–NiCo–ZIF	TiO_2_–NiCoS–PC	Core/shell	NiCoS–PC	UV–vis 300 W Xe lamp	1.29	^[^ ^117^ ^]^
SiO_2_@HKUST‐1‐Ti	Cu–TiO_2_/C	Hollow nanospheres	Pt	Simulated sunlight 300 W Xe lamp	14.05	^[^ ^100^ ^]^
UiO‐66‐NH_2_/g‐C_3_N_4_	C‐ZrO_2_/g‐C_3_N_4_/Ni_2_P	Nanosheet	EY and Ni_2_P	>420 nm 300 W Xe lamp	10.04	^[^ ^101^ ^]^
MIL‐100(Fe)/g‐C_3_N_4_	g‐C_3_N_4_/*α*‐Fe_2_O_3_	Nanosheet	Pt	>420 nm 300 W Xe lamp	2.07	^[^ ^134^ ^]^
NiCo‐MOF@g‐C_3_N_4_	g‐C_3_N_4_–NiCoP_2_–PC	Nanosheet	–	UV–vis 300 W Xe lamp	2.1	^[^ ^133^ ^]^
g‐C_3_N_4_/Al‐MOF	Cd(OH)_2_/CdS/g‐C_3_N_4_/NPC	Nanosheet	–	Simulated sunlight 150 W Xe lamp	0.15	^[^ ^141^ ^]^
rGO/ZIF‐8	ZnS/rGO/CuS	Nanosheet	–	>420 nm 300 W Xe lamp	2.61	^[^ ^132^ ^]^
Ni‐MOF@g‐C_3_N_4_	Ni@g‐C_3_N_4_	Nanosheet	EY	>420 nm 300 W Xe lamp	2.99	^[^ ^136^ ^]^
Fe/MIL‐125	Fe/TiO_2_@C	Nanodisc	–	UV–vis 500 W Xe/Hg lamp	215	^[^ ^142^ ^]^
NH_2_‐MIL‐101(Fe)/Ni(OH)_2_/g‐C_3_N_4_	C–N/Fe_2_P/Ni_2_P/g‐C_3_N_4_	Nanoshuttle	EY and Ni_2_P	>420 nm 300 W Xe lamp	13.81	^[^ ^76^ ^]^
NH_2_‐MIL‐125	N–C–TiO_2_/C	Nanodisc	–	UV–vis 500 W Xe/Hg lamp	0.43	^[^ ^143^ ^]^
NH_2_‐MIL‐125(Ti/Cu)	TiO_2_/Cu*_x_* O/C	Nanodisc	–	UV–vis 500 W Xe/Hg lamp	3.3	^[^ ^144^ ^]^

#### 4.1.4 3D Nanocomposites

Compared to the low‐dimensional (0D, 1D, and 2D) MOF templates, it is relatively easy to synthesize 3D MOFs precursors. The 3D MOF precursors offer great flexibility of choices for template materials, chemical compositions and morphologies, which possess much higher surface area, tailorable pore sizes and easy hydrophilic/hydrophobic surface functionalization.^[^ ^17^ ^]^ In particular, 3D MOF precursors can host a variety of guest species to derive unlimited nanocomposites that combine the conductive porous carbons and metallic/multimetallic oxides, sulfides, phosphides, carbides and alloy nanoparticles with engineered energy bandgaps, which can potentially absorb a major part of sunlight in the UV–vis–IR range for efficient heterostructured photocatalytic applications.^[^ ^11^, ^20^, ^36^, ^48^, ^71^, ^145^ ^]^ The MOF derived nanocomposites with desirable morphologies and chemical properties can be readily replicated from the rationally designed MOF precursors. A variety of morphologies such as cubes, discs, spheres and sponges can be easily obtained by the direct pyrolysis of the 3D MOFs precursors.

##### Nanocubes

The morphologies and crystallite sizes of the MOF precursors can be adjusted via a wet‐chemical approach by adjusting the pH values and the concentrations of the solvents and reactants. The 3D cubic MOF precursors such as ZIF‐67, ZIF‐8 and UiO‐66 can easily be synthesized via the modified solvothermal method.^[^ ^43^, ^146^ ^]^ Nanocubic composites derived from these cubic MOF precursors exhibit higher BET surface area and exposed active sites, which can improve photocatalytic performance. However, as discussed in previous sections, MOF derived single‐metal oxide/carbon composites only show moderate catalytic activities due to the limitations of light absorption and the poor charge generation/transport.^[^ ^147^ ^]^ Therefore, it is important to synthesize multimetallic MOF derived nanocomposites coupled with noble metal‐free cocatalysts to form heterojunctions for improved H_2_ evolution under visible light irradiation. For example, NH_2_‐UiO‐66 (Zr) was mixed with melamine derived g‐C_3_N_4_ by self‐assembly mechanism under ultrasonication, followed by heat treatment at 600 °C first in an inert atmosphere then in air to obtain 3D cubic C, N‐codoped ZrO_2_/g‐C_3_N_4_ nanocomposites. Finally, different wt% of Ni_2_P as a cocatalyst was deposited to obtain C, N‐codoped ZrO_2_/g‐C_3_N_4_/Ni_2_P nanocomposite.^[^ ^101^ ^]^ This external‐templated MOF derived cubic C, N–ZrO_2_/g‐C_3_N_4_/Ni_2_P nanocomposite showed exceptionally high photocatalytic H_2_ evolution rate of 10.04 mmol g_cat_^−1^ h^−1^ under visible light and apparent quantum yield of 35.5% at 420 nm. The dramatically improved H_2_ evolution activity was attributed to the enhanced light absorption and spatial separation of photogenerated charge carriers due to the formation of staggered band heterojunction resulting from the narrow energy bandgap of C, N‐codoped ZrO_2_ with the semiconducting g‐C_3_N_4_ (type II heterostructure). Furthermore, the highly conductive g‐C_3_N_4_ and the uniformly anchored Ni_2_P cocatalyst accelerated the charge transfer and the surface photocatalytic reactions, respectively.^[^ ^101^ ^]^

##### Nanodiscs

**Figure 6:**
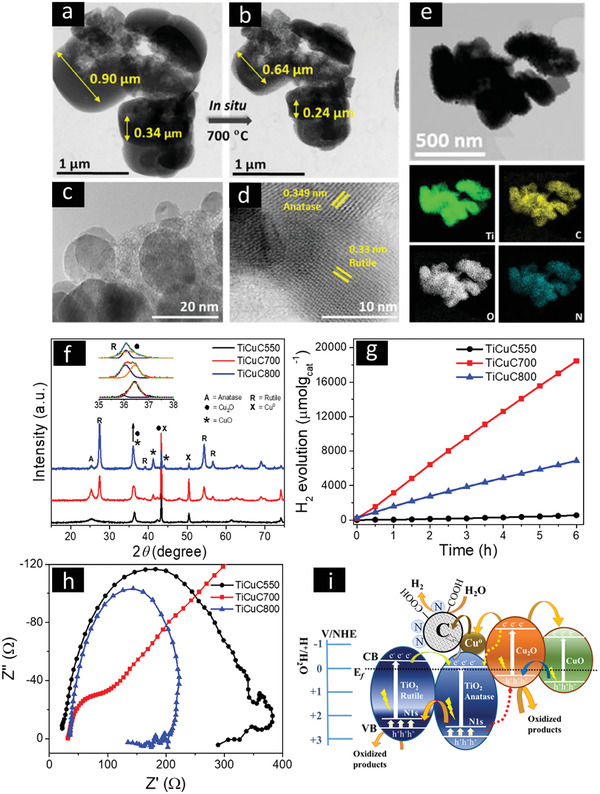
STEM image of as‐prepared a) NH_2_‐MIL‐125(Ti) and b) after in situ heating under N_2_ gas atmosphere up to 700 °C. The shrinkage of particle size (≈30% volume) can be observed with the preserved morphology and porosity. Reproduced with permission.^[^ ^149^ ^]^Copyright 2021, Elsevier. c,d) HRTEM images and e) EDX elemental maps of NH_2_‐MIL‐125(Ti) derived composite N–C–TiO_2_/C_ArW_. c‐e) Adapted with permission.^[^ ^143^ ^]^ Copyright 2021, Elsevier. f) PXRD patterns, g) photocatalytic H_2_ evolution performance, and h) electrochemical impedance spectra (EIS) of Bi‐MOF, NH_2_‐MIL‐125(Ti/Cu) derived composites TiO_2_/Cu*_x_* O/C (named as TiCuC) at 550, 700, and 800 °C. i) Schematic illustration of the proposed mechanism of charge generation/migration and photocatalytic H_2_ evolution over NH_2_‐MIL‐125(Ti/Cu) derived multiheterojunction TiCuC700 under UV–vis light. f‐i) Adapted with permission.^[^ ^144^ ^]^ Copyright 2021, Royal Society of Chemistry.

One of the most stable Ti‐MOF, self‐templated MIL‐125 can be pyrolyzed under the inert gas atmosphere to derive TiO*_x_*/C nanocomposites with disc‐like 3D morphologies. Benefiting from the structural stability of Ti‐MOF, various transition metal guest species, such as Fe, Ni, Co, Cu, Zn, Ag and Au, can be loaded onto the as‐prepared MIL‐125 precursor by impregnation method.^[^ ^63^, ^64^ ^]^ Moreover, Ti‐MOFs can also be coupled/grown on external‐template carbon sources (such as g‐C_3_N_4_) to obtain morphologically unique metal oxide/carbon composites.^[^ ^148^ ^]^ Bimetallic compound/carbon composites can be easily formed via pyrolysis of the transition metal loaded Ti‐MOF at high temperature. For example, Valero‐Romero et al. demonstrated that the carbonization of Fe impregnated MIL‐125(Ti) above 500 °C in inert atmosphere produced Fe‐doped TiO_2_ nanoparticles embedded in amorphous carbon matrix with inherited 3D disc‐like morphology. Furthermore, by controlling the pyrolysis conditions of MIL‐125, the phase ratios of polymorph TiO_2_ with tunable crystalline phases (anatase and rutile) in the derived nanocomposites could be in situ optimized to achieve higher photocatalytic performance. The porous carbon matrix kept the TiO_2_ nanoparticles from agglomerations, which resulted in uniform dispersion of polymorph TiO_2_ for enhanced light absorption and better exposed active sites. The Fe‐doped mixed‐phase (anatase/rutile) TiO_2_/C nanocomposite obtained by pyrolysis of 0.15 wt% Fe loaded MIL‐125 at 700 °C showed photocatalytic H_2_ activity of 215 µmol g_cat_^−1^ h^−1^ under UV–vis light.^[^ ^142^ ^]^ Recently, our group demonstrated that self‐templated amino‐functionalized NH_2_‐MIL‐125(Ti) derived nanocomposites under water vapor atmosphere exhibit even better photocatalytic H_2_ activity. As shown in **Figure** **6**, pyrolysis of NH_2_‐MIL‐125(Ti) at 700 °C under a controlled water vapor atmosphere produces N and carboxyl group decorated porous carbon with uniformly embedded N/C‐codoped TiO_2_ nanoparticles. Introducing water vapor as a mild oxidizing agent during the pyrolysis forms additional oxygen‐rich N like interstitial/intraband states lying above the valence band of TiO_2_ along with the self‐doped carbon, which further narrows the energy bandgaps of polymorphic TiO_2_ nanoparticles that enhance photocatalytic charge transfer efficiency. This NH_2_‐MIL‐125(Ti) derived sample (N–C–TiO_2_/C_ArW_) at 700 °C under water vapor exhibited 426 µmol g_cat_^−1^ h^−1^ under UV–vis light without any cocatalyst.^[^ ^143^ ^]^ Although the surface functionalization and the formation of TiO_2_ phasejunction improved the photocatalytic H_2_ evolution performance compared to the pure TiO_2_, the formation of optimized p–n heterojunction between different metal oxides can further improve the photogeneration and migration of electrons and holes, therefore further enhance the photocatalytic performance. The bi‐MOF, NH_2_‐MIL‐125(Ti/Cu) was used as sacrificial template to derive multiheterostructured TiO_2_/Cu*_x_* O/C nanocomposites. One‐step direct pyrolysis of NH_2_‐MIL‐125(Ti/Cu) in water vapor at optimal temperature of 700 °C results in the formation of a p–n heterojunction between TiO_2_ and Cu*_x_* O nanoparticles. In this method, concurrently, a phasejunction between nitrogen/carbon codoped anatase and rutile TiO_2_ is formed, accompanied with the formation of Cu*_x_* O heterostructures. These multiheterostructures are embedded in N‐containing and hydrophilic carboxyl functionalized carbon matrix. These optimized TiO_2_/Cu*_x_* O/C multiheterostructural composites absorbed more visible light, offered multiple pathways for migration of photoinduced electrons and holes, and provided increased number of active sites for photocatalytic reactions. Without loading expensive noble metals, TiO_2_/Cu*_x_* O/C nanocomposites derived at 700 °C exhibited photocatalytic H_2_ generation activity of 3298 µmol g_cat_^−1^ h^−1^ under UV–vis light.^[^ ^144^ ^]^ This simple method can be extended to prepare other metal/metal oxide/carbon 3D nanocomposites with high BET surface area, controllable crystal sizes, and tunable chemical phases and compositions from metal/multimetal‐doped MOFs for enhanced photocatalytic H_2_ evolution. Particularly, studies to explore rationally designed 3D MOF derived nanocomposites with diverse morphologies such as nanocages, nanospheres, nanoarrays, honeycomb structures and nanofibers for visible light photocatalysis are highly desirable.^[^ ^39^ ^]^

#### 4.1.5 Summary of MOF Derived Nanocomposites for Photocatalytic H_2_ Evolution

The photocatalytic H_2_ evolution performance of semiconductor materials mainly depends upon the appropriate energy band position for light absorption, the charge generation/migration and the accessible active sites. Moreover, the high BET surface area of the MOF derived metal compound/carbon composites with various dimensions and morphologies also play important role in better light absorption and improved catalytic activity by preventing catalyst particle agglomeration and providing well exposed active sites. Also, it is worth mentioning that the energy band positions of metal compounds (oxides, sulfides or phosphides) in MOF derived composites can be in situ tuned by doping heteroatoms, creating phase/heterojunctions between metal–metal or metal–carbon compounds to enhance charge migration and suppress charge recombination. Loading non‐noble cocatalysts in MOF derived composites can decorate the porous carbon matrix providing additional active sites for highly efficient photocatalytic performance. In MOF derived nanostructures, photogenerated charge flow can be regulated in a specific direction in 1D and 2D nanocomposites for improved photocatalytic performance. However, only a few 2D external‐templated MOFs with g‐C_3_N_4_ and rGO derived nanocomposites are reported due to the difficulty in morphology control. More efforts need to be dedicated to the synthesis and in‐depth investigation of MOF derived composites with optimal semiconducting and catalytic properties to achieve viable highly efficient photocatalysts for H_2_ evolution.

### 4.2 MOF Derived Nanocomposites for Photodegradation of Organic Pollutants

The presence of alarmingly high amounts of toxic industrial organic pollutants is one of the greatest challenges to our environment and society. MOF derived composites have widely been explored for the photodegradation of organic pollutants such as textile dyes, pharmaceutical contaminants, phenols and antibiotics.^[^ ^84^, ^110^, ^150^, ^151^ ^]^ The MOF derived composites with surface‐functionalized porous carbons offer high BET surface area and pore volume with appropriate pore size for the adsorption of pollutant molecules. The adsorbed pollutant molecules are simultaneously photodegraded into environmentally benign species by the metal oxides, sulfides or phosphides with optimized energy bandgaps embedded in the MOF derived porous carbon matrix.^[^ ^47^ ^]^ For maximum photodegradation efficiency, along with other properties, it is also important to have matched energy levels of pollutant (*E*_LUMO_) and photocatalyst (*E*_VB_). For instance, frontier orbital energies (HOMO–LUMO gap) between *E*_HOMO_ and *E*_LUMO_ of some model organic pollutants are as followings: methylene blue (MB): 2.49 eV; rhodamine‐B (RhB): 2.84 eV; rhodamine‐640 (Rh640): 2.74 eV; phenol: 0.51 eV.^[^ ^102^, ^103^ ^]^ The easiness of modifiable energy bandgaps of MOF derived composites can offer a better strategy to match the degradation energy levels of the pollutants. In 2010, Yang et al. reported that 3D cubic ZnO@C composite, which was derived from MOF‐5 under N_2_ atmosphere at 600 °C, exhibited BET surface area of >500 m^2^ g^−1^ and demonstrated higher adsorption and photocatalytic degradation of RhB under UV irradiation.^[^ ^62^ ^]^ Since then, much effort has been made to produce MOF derived nanocomposites with a variety of morphologies for photodegradation of organic pollutant applications. These nanocomposites can be decorated with hydrophilic/hydrophobic functional groups and doped with heteroatoms (metals and/or nonmetals) to achieve the desired properties. Moreover, complicated nanocomposites including multimetallic oxides, sulfides and phosphides embedded in in situ formed porous carbon matrix can be generated from selected MOFs or MOF‐based composites.^[^ ^75^, ^84^, ^130^, ^152^, ^153^, ^154^ ^]^ In the following section, the relationship of structure/morphologies between MOF precursors and the derived nanocomposites for photodegradation of organic pollutants are presented. Moreover, the effects of the semiconducting and physicochemical properties of these derived nanocomposites on their photocatalytic applications are also analyzed and summarized.

#### 4.2.1 0D Nanocomposites

##### Polyhedra

It is highly desirable to find materials that can not only show high adsorption capacities but also simultaneously photodegrade the adsorbed species into neutral products with high efficiency. Zhang et al. reported that MIL‐53(Fe) derived *γ*‐Fe_2_O_3_/C nanoparticles with polyhedral morphologies possessing a surface area of 397 m^2^ g^−1^ were more efficient for dye adsorption and photodegradation (863 mg g^−1^) in the presence of a scavenger H_2_O_2_.^[^ ^155^ ^]^ Other magnetic nanocomposite such as Co/CoO nanoparticles embedded in the NPC matrix with rhombic dodecahedral morphology can be derived from one‐step direct carbonization of ZIF‐67 under N_2_ at 800 °C.^[^ ^110^ ^]^ The highly crystalline Co/CoO nanoparticles are spatially distributed in the NPC matrix with a high BET surface area of 345 m^2^ g^−1^ and open network of micro/mesopores which facilitate the fast diffusion of organic pollutant MB molecules. However, despite the strong magnetic response and high adsorption capabilities (500 mg g^−1^) of MB molecules due to the *π*–*π* interaction between the MB molecules and the graphitic carbon, the photodegradation performance of this materials tends to be poor.^[^ ^110^ ^]^ Though FeO*_x_* and CoO*_x_* nanoparticles are easy to collect after the completion of photocatalytic reactions due to their magnetic properties, their photoresponse is generally poor compared to the ZnO and TiO_2_ semiconducting nanoparticles.

The direct carbonization of MOFs with polyhedral morphologies (such as MOF‐5, ZIF‐8 and ZIF‐67) in the inert gas atmosphere above 500 °C results in metal or metal oxide/carbon composites. The morphologies and particle sizes of the MOFs precursor can be tuned by the selection of synthesis method and the reaction conditions such as molar ratio and concentration of the reactants, type of solvents, synthesis temperatures and duration of the reaction.^[^ ^21^, ^42^ ^]^ These multiedge and multifacet composites offer numerous accessible catalytic active sites and interconnected open pores with tunable pore diameters. Zhang et al. reported octahedral porous ZnO@C nanocomposites (with the energy bandgap of 2.92 eV) derived from MOF‐5 at different carbonization temperatures for photocatalytic degradation of RhB under visible light.^[^ ^156^ ^]^ However, pure ZnO and TiO_2_ semiconducting nanoparticles show limited photocatalytic performance due to their relatively large energy bandgaps (>3.2 eV). To overcome this limitation, the presence of heteroatoms such as C, N, S and P (either self‐doped or introduced as a guest species) in the MOF derived nanocomposites can narrow the energy bandgaps and enhance the visible light absorption capability. Liang et al. has reported the fabrication of C/N‐doped ZnO nanoparticles with a narrowed energy bandgap of 2.98 eV embedded in porous carbon matrix by a two‐step thermal conversion of ZIF‐8 (N‐rich and Zn‐containing) at 600 °C.^[^ ^75^ ^]^ These polyhedral ZnO/porous carbon composite show superior MB adsorption and photodegradation capacity under the simulated sunlight due to the higher number of active sites, N/C dopants and oxygen vacancies. Also, the presence of hydrophilic functional groups attached to the surface of the carbon matrix can further enhance the adsorption of MB on the carbon matrix. Further, the carboxylate functional groups (—COOH/—OH) can be easily introduced by carbonizing MOFs in a controlled water vapor atmosphere at higher temperatures. For instance, the carbonization of ZIF‐8 at 800 °C under water vapor as a mild oxidizing agent results in the atomically homogeneous distribution of N‐doped ZnO nanoparticles embedded in —COOH functionalized porous carbon matrix with roughly rhombic dodecahedron morphology. The resulting ZnO/C nanocomposite with high BET surface area (up to 995 m^2^ g^−1^), large pore volume (up to 0.58 cm^3^ g^−1^) and excessive oxygen‐containing hydrophilic functional groups exhibit stronger interaction between the porous carbon and the MB molecules and facilitates the simultaneous adsorption and photodegradation of MB under visible light.^[^ ^84^ ^]^

**Figure 7:**
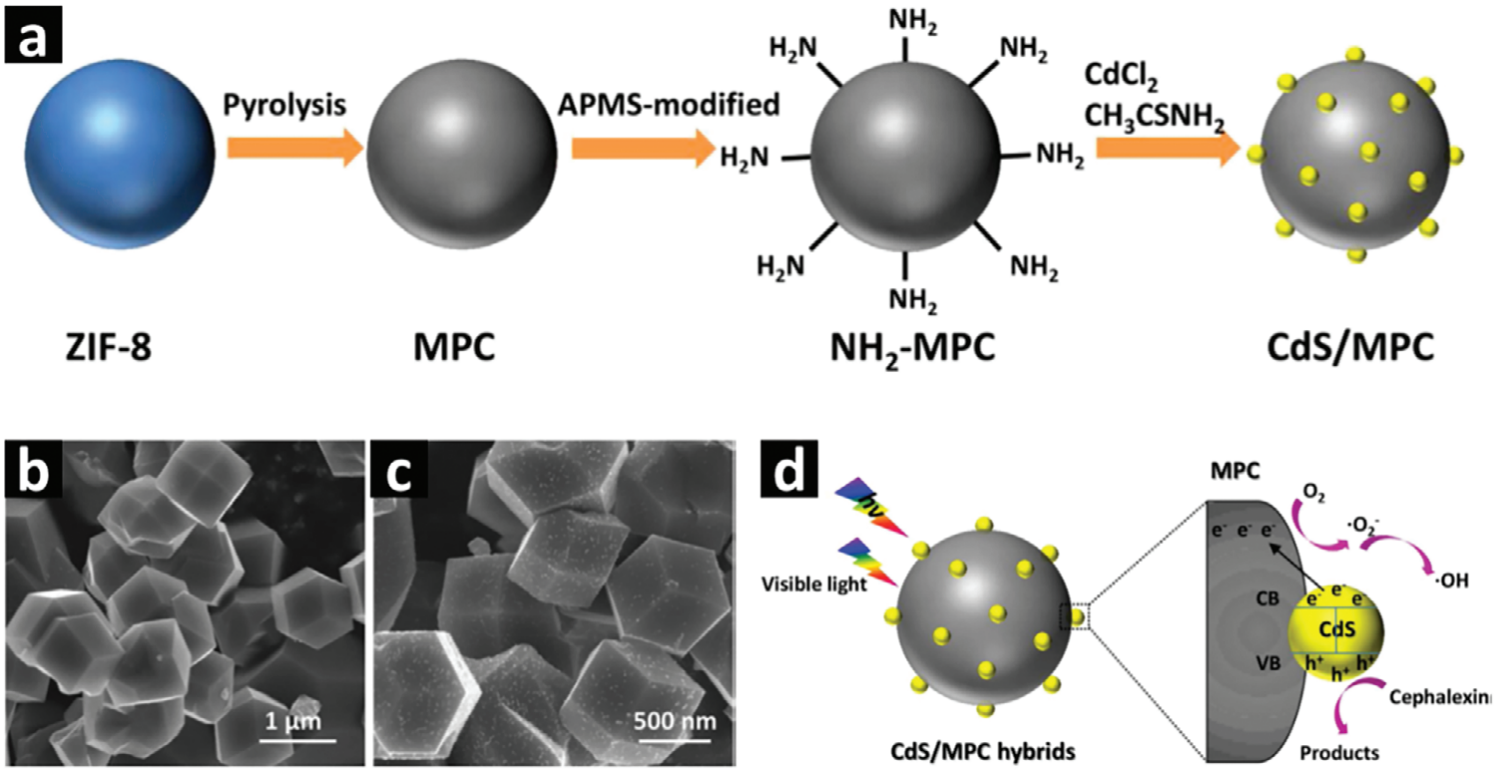
a) Schematic drawing of the synthesis process of the CdS/MPC nanocomposites. b) SEM images of ZIF‐8 precursor. c) SEM of ZIF‐8 derived 20 wt% CdS/MPC nanocomposite. d) Schematic illustration of the photocatalytic mechanism over the CdS/MPC nanocomposite. Adapted with permission.^[^ ^150^ ^]^ Copyright 2017, Elsevier.

A variety of doped metal oxides embedded in polyhedral carbon matrix can be derived from thermally stable MOFs. For example, octahedral P‐doped Cu_2_O/C composite can be prepared by pyrolysis of the modified HKUST‐1 at a moderate temperature of 300 °C. This nanocomposite shows the relatively narrow energy bandgap (2.04 eV) and exposed active sites present in the porous carbon matrix and exhibits superior photodegradation of phenol under visible light irradiation due to the better sunlight absorption capability.^[^ ^151^ ^]^ Among the other semiconductors with a narrow energy bandgap, CdS is a prominent photocatalyst with a bandgap of 2.4 eV. However, the easy agglomeration and fast charge recombination of CdS nanoparticles restrict their photocatalytic activity. This issue can be tackled by using MOF as a sacrificial template to produce CdS/carbon composites with uniformly dispersed CdS nanoparticles by high‐temperature carbonization. As shown in **Figure** **7**, first polyhedral ZIF‐8 precursor was employed to derive N‐rich mesoporous carbon (MPC) with large surface area of 933 m^2^ g^−1^ and pore volume of 0.64 cm^3^ g^−1^. Then optimized CdS/MPC composites were obtained by loading different wt% of CdS for photodegradation of antibiotics and other pharmaceutical contaminants.^[^ ^150^ ^]^ Compared to the composite obtained by the conventional physically mixed method, this MOF derived composite offers good distribution of semiconducting nanomaterials in carbon matrix preventing agglomerations, reduced charge recombination, and enhanced adsorption and photodegradation capability.

##### Self‐Templated Core–Shell Nanostructures

**Figure 8:**
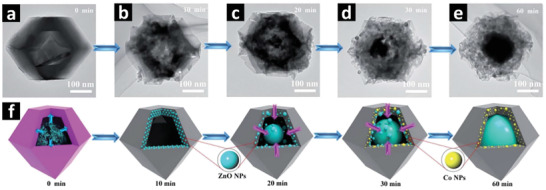
a–e) TEM images of the thermal transformation of Zn/Co–ZIF into ZnO@C–N–Co nanocomposites at different carbonization times and f) schematic representation of the formation of the core–shell ZnO@C–N–Co. Reproduced with permission.^[^ ^111^ ^]^ Copyright 2017, Royal Society of Chemistry.

The core–shell nanocomposites offer synergistic effects between the core and the shell components of the nanocomposites with enhanced structural stability, tunable functionalities and good dispersibility, consequently improved photodegradation performance. The core–shell nanostructures can be usually derived from the predesigned bimetallic self‐templated MOF precursors. For example, one‐step direct carbonization of the as‐synthesized Fe_3_O_4_@HKUST‐1 in N_2_ at 500 °C can readily transform the bimetallic MOF precursor into Fe_3_O_4_ core encapsulated in the carbon shell (≈50 nm thick) decorated with Cu nanoparticles. The thickness and porosity of the carbon shell around the metal oxide core can be easily tuned by controlling the pyrolysis conditions. This MOF derived Fe_3_O_4_@C/Cu core–shell nanostructure shows good absorption of visible light due to the narrow energy bandgap of 1.97 eV. Moreover, the magnetic response of the Fe_3_O_4_ core makes these core–shell nanocomposites easy to collect after the photocatalytic reaction. Though this Fe_3_O_4_@C/Cu core–shell nanocomposite outperforms the commercial TiO_2_ and g‐C_3_N_4_ in photodegradation of MB, H_2_O_2_ needs to be added to enhance the production of active oxygen species for the photodegradation of MB.^[^ ^93^ ^]^ To overcome the limitations of low photocatalytic performance of Fe_3_O_4_@C/Cu core–shell nanostructure, Ti‐ or Zn‐based MOF precursor can be employed to form metal oxide core. Using self‐templated precursor, bimetallic Zn/Co‐MOF composite with Zn–ZIF‐8 as the shell and Co–ZIF‐67 as the core, ZnO@C–N–Co core–shell nanocomposite was synthesized by the direct pyrolysis of the as‐prepared Zn/Co–ZIF at 600 °C for 2 h under argon atmosphere. The time‐dependent pyrolysis observed by TEM shown in **Figure** **8** reveals that Zn^2+^ in the ZIF‐8 shell is converted into ZnO NPs, which are uniformly distributed in the amorphous carbon shell within the first 10 min of the pyrolysis. With the progression of pyrolysis, ZnO NPs left the mesoporous carbon shell, migrated to the hollow interior cavity and aggregated to produce larger ZnO particles, whereas Co ions were in situ reduced to Co NPs by the formed carbon and migrated inversely from the hollow interior to the ligand‐derived N‐doped graphitized carbon shell. The highly synergistic effect between the robust ZnO core and the C–N–Co shell in the derived ZnO@C–N–Co core–shell nanocomposite demonstrates remarkable photocatalytic degradation of methylene orange (MO). Moreover, the uniform distribution of Co NPs on the mesoporous C shell facilitates easy separation of photocatalysts by using an external magnet.^[^ ^111^ ^]^ Based on this work, various core–shell multimetal oxide/carbon composites can be expected to be produced via the pyrolysis of rationally designed self‐templated hollow MOF precursors.

##### External‐Templated Core–Shell Nanostructures

It is a persisting challenge to achieve scalable production of MOF derived core–shell nanocomposites with morphological uniformity and high efficiency. Preparation of core–shell nanostructures obtained by one‐step direct pyrolysis of self‐templated MOF works only for a few MOF precursors. Most importantly, it remains a great challenge to precisely control the thickness and reproducible porosities of the MOF derived carbon shells. Alternative approach such as external‐templated MOF derived nanocomposites may offer the solutions to overcome this problem. For example, ZIF‐8 was first grown on the surface of rGOs, followed by the calcination of the as‐synthesized ZIF‐8/rGO in air at 500 °C. The ZIF‐8 precursor decomposed at above 400 °C in air to release C and N species in the form of CO_2_ and NO*_x_* and left behind the ZnO nanoparticles encapsulated in the rGO shell of less than 10 nm thickness. The thickness of the rGO shell and the weight percentage of ZnO/rGO were optimized by tuning the ratios of ZIF‐8 and rGO precursors. These external‐templated‐MOF derived composites show good photocatalytic activity for the degradation of contaminant Orange II under simulated sunlight irradiation. The rGO shell traps the photogenerated electrons and enhances the conductivity of the composite, resulting in reduced charge recombination and consequently improved photodegradation activity.^[^ ^113^ ^]^ Compared to the self‐templated MOF derived core–shell structures, in general, these external‐templated metal oxide/rGO core–shell structures offer lower BET surface area that limit their adsorption capacities. So far, there are only very limited studies published on MOF derived core–shell nanostructures for photocatalytic applications.^[^ ^93^, ^111^, ^113^, ^157^ ^]^ More investigations need to be carried out to find new combinations of MOF derived core–shell nanostructures with high surface area that contains not only metal oxides but also other metal compounds such as metal sulfides and metal phosphides.

#### 4.2.2 1D Nanocomposites

##### Nanorods

1D nanocomposite with nanorod morphology can be synthesized by employing self‐templated Fe‐MOF precursor. For example, MIL‐88A can be carbonized at 600 °C under N_2_ atmosphere to derive Fe_2_O_3_ nanoparticles uniformly distributed in a rod‐like porous carbon matrix, making it magnetic porous adsorbent for decolorization of RhB dye from wastewater. Though Fe_2_O_3_ nanoparticles make this composite easily collectable due to the magnetic property, their photoresponse is not as good as semiconducting ZnO or TiO_2_ nanoparticles. It is necessary to add additional oxidants such as peroxides and/or persulfates with the magnetic Fe_2_O_3_/C photocatalyst to generate a combined catalytic effect for photodegradation of the RhB under UV light.^[^ ^112^ ^]^ Despite the limitation, this nanocomposite shows structural stability and good recyclability to remove RhB from wastewater.

Apart from metal oxide/C composites, it is important to explore new efficient visible‐light photocatalysts. In this respect, cadmium sulfate (CdS) can be a suitable photocatalyst owing to its narrow energy bandgap (2.4 eV) that guarantees the photoactivity under visible light irradiation. However, CdS‐based semiconductors are prone to photo corrosion in aqueous medium, which leads to the potential release of toxic Cd^2+^ ions. This issue can be tackled by combining the CdS semiconductor particles with carbon materials, which function not only as stable support to effectively distribute the CdS particles but also offering higher surface area for the adsorption of the contaminants. Self‐templated Cd‐MOF can be an excellent precursor for the in situ derivation of N‐doped carbon‐supported CdS nanocomposites with nanorod morphologies for visible‐light‐driven photodegradation of antibiotics. Recently, Cao et al. reported CdS/NC nanorods derived from direct carbonization of Cd‐MOF at various temperatures (400–700 °C) under an inert atmosphere. They demonstrated that CdS/NC derived at 500 °C showed the highest photodegradation (83%) of Tetracycline (TC) antibiotic in 1 h under visible light. The improved performance of photodegradation of TC was ascribed to the efficient transportation of photogenerated charge (e^−^/h^+^) carriers due to the enhanced interfacial interactions of CdS nanoparticles uniformly distributed in the N‐doped carbon matrix.^[^ ^158^ ^]^

##### Nanotubes

**Figure 9:**
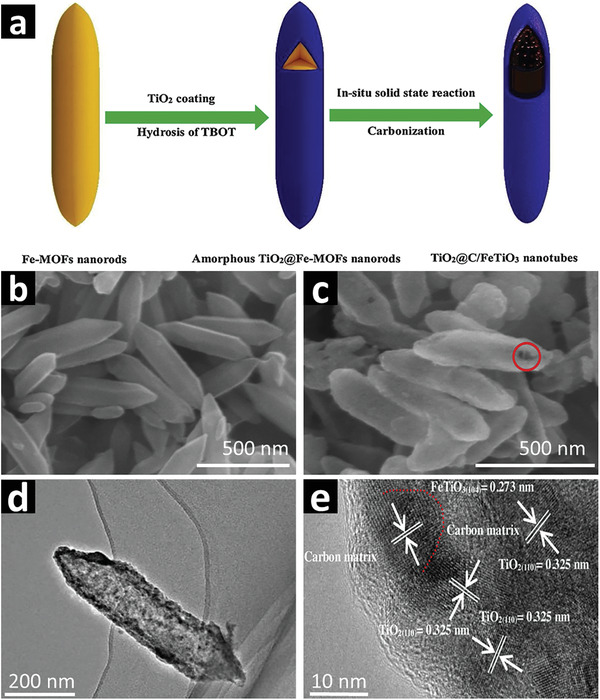
a) Schematic diagram of the carbonization process of hollow TiO_2_@C/FeTiO_3_ nanotubes. b) FESEM image of Fe‐MOF precursor. c) TCF‐25% nanotubes and d) TEM images of TCF 20%. Adapted with permission.^[^ ^159^ ^]^ Copyright 2019, Elsevier.

A very few studies are available on self‐templated MOF precursor derived 1D hollow nanocomposites with well‐defined morphologies for photocatalytic applications. One of the main challenges in MOF derived composites is to strictly produce tailored porosities and atomic ratios of metals, metal oxides and functionalized porous carbon components.^[^ ^39^ ^]^ If the distribution of metal or metal oxide nanoparticles in porous carbon matrix are optimized, the obtained nanocomposites can exhibit exceptionally high photocatalytic performance due to the improved photoinduced electric charge generation and the efficient photoelectrochemical reactions. For example, TiO_2_ coated MIL‐88(Fe) precursor with nanorod morphology has been directly carbonized at 600 °C under N_2_ atmosphere for a few hours to obtain respective hollow ternary TiO_2_@C/FeTiO_3_ nanocomposites with different Ti/Fe molar ratios. These one‐step MOF derived novel bimetallic nanocomposites possess hollow nanotube morphology, in which the TiO_2_ layer was deposited onto the surface of FeTiO_3_, embedded in an inner porous carbon layer. Such bimetal oxide/carbon hollow nanostructures can maximize the sunlight absorption for efficient photoelectrochemical degradation of phenol. Wang et al. demonstrated in **Figure** **9** that the wall thickness of the TiO_2_ layer (20 to 25 nm) can be tuned by adjusting the molar ratio of Fe and Ti species between 15% and 25%. This self‐templated multimetallic MOF precursor strategy offers the flexibility to regulate the structural, textural and morphological features related to their photocatalytic properties. The photoelectrochemical efficiency is improved due to the synergistic effects between the middle conductive layer, suitable energy bandgaps, full‐spectrum light absorption and electro‐oxidation assisted photocatalysis.^[^ ^159^ ^]^

##### Nanoshuttles

As discussed in Section 4.1.2, photocatalytically active 1D metal oxide/C nanocomposites can be prepared by a method such as physical and chemical mixing of metal oxides with carbonaceous matrix (e.g., carbon nanotubes, rGO or graphene nanosheets).^[^ ^160^, ^161^ ^]^ Self‐templated MOF precursors can offer the flexibility of a “two‐for‐one” approach to directly carbonize the as‐synthesized MOFs to yield 1D metal oxide/carbon nanocomposites with high surface area and accessible chemically reactive sites. Recently, Pang et al. reported the successful synthesis of *α*‐Fe_2_O_3_/C nanoshuttles via direct pyrolysis of the self‐templated Fe‐MIL‐88B precursor at 550 °C for 2 h in argon. Upon carbonization, the hexagonal prism (with a truncated hexagonal cone at each end) shaped Fe‐MIL‐88B crystals with an average size of 1.8 µm were transformed into shuttle‐like *α*‐Fe_2_O_3_/C nanocomposite with mesoporosity and uniform elemental distribution. These *α*‐Fe_2_O_3_/C nanoshuttles demonstrated superior photocatalytic activity toward the photodegradation of MB under simulated sunlight, due to the effective separation and fast migration of photogenerated charges on *α*‐Fe_2_O_3_ as well as the relatively large number of exposed chemical reaction active sites on the mesoporous carbon matrix.^[^ ^162^ ^]^

Currently, self‐templated MOF derived nanocomposites are dominantly reported in the literature for photodegradation applications. Using external‐templated methods, rationally designed new 1D MOF structures with well‐controlled morphologies could be synthesized to derive 1D nanocomposites for highly efficient and stable photocatalysts for photodegradation of organic and industrial pollutants.

#### 4.2.3 2D Nanocomposites

##### Nanoplatelets

Recently, Lin et al. reported that a square‐shaped 2D Cu‐MOF precursor with lateral dimensions of 4–6 µm and average thickness of 80 nm was prepared through a classic solvothermal treatment of metal node Cu(NO_3_)_2_, organic ligand 4,4′‐bipyridine, and surfactant polyvinyl pyrrolidone (PVP) in ethanol under 100 °C. The PVP acted as an “inhibitor” preventing the layer stacking growth and directing the coordination of Cu metal nodes with the linear ligand 4,4′‐bipyridine.^[^ ^163^ ^]^ Controlling the pyrolysis conditions such as temperature and gas atmosphere is critical to deriving the Cu_2_O/C composite with 2D morphology and high BET surface area. When the Cu‐MOF precursor decomposed at higher temperatures (above 500 °C) under argon, Cu species transformed into Cu^0^ and diffused to the surface of the carbon matrix. At relatively lower temperature (below 300 °C) under argon, the 2D morphologies of the MOF precursor can be retained. The BET surface area of the 2D MOF precursor and the derived Cu_2_O/C square nanoplatelets is 7.4 and 109.5 m^2^ g^−1^, respectively. The interesting 15‐fold increase in BET surface area with preserved square‐like nanoplatelet morphology was attributed to the flexibility of the carbon matrix with slit‐like pores. These 2D Cu_2_O/C square nanoplatelets showed superior photodegradation rate (2.5 mg min^−1^ g_cat_^−1^) of MO under visible light compared to other Cu_2_O/C‐based photocatalysts.^[^ ^163^ ^]^ So far, only a few self‐templated 2D MOF derived metal oxide/carbon composites have been reported due to the challenges of controlling the morphologies of the self‐assembled MOF precursors. To overcome this challenge, 2D external templates such as carbon structures can be used to direct the growth of MOF precursors.

##### External‐Templated Nanosheets

**Figure 10:**
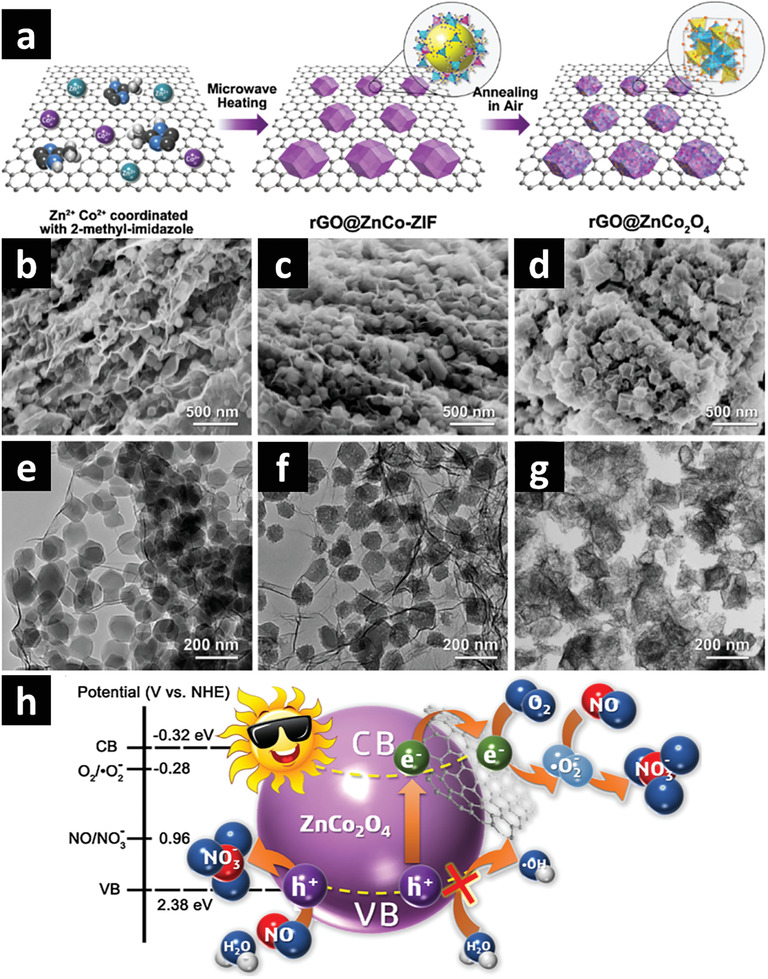
a) Schematic diagram of the formation of the rGO@ZnCo_2_O_4_. SEM images of b) rGO–ZnCo–ZIF precursor, c) nanocomposite GZC‐300, and d) GZC‐500. TEM images of e) rGO–ZnCo–ZIF precursor, f) GZC‐300, and g) GZC‐500. h) Image of the photocatalytic mechanism of NO oxidation by GZC‐300 under visible‐light. Adapted with permission.^[^ ^130^ ^]^ Copyright 2018, Elsevier.

In an early attempt, MOF derived metal oxides (ZnO) were combined with rGO by the microwave‐assisted method to generate synergic effect between the ZnO nanoparticle with semiconducting property and the rGO with high light absorption property for photodegradation of MB.^[^ ^129^ ^]^ Though this ZnO/rGO nanosheet showed improved photodegradation performance compared to pristine ZnO, it is challenging to obtain homogeneous loading of ZnO on rGO nanosheets. The 2D nanostructures such as graphene, GO or rGO can be employed as an external template to grow MOFs, and the resulting MOF/2D carbon nanostructures possess combined properties of the porous MOF and the conductive external template. For example, Zn–Co–ZIF can be directly grown on GO assisted by PVP as a capping agent. As shown in **Figure** **10**, rhombic dodecahedron shaped Zn–Co–ZIF crystals (≈150 nm) are uniformly dispersed on exfoliated rGO wrinkled nanosheets. Heat treatment of this bimetallic rGO/ZnCo–ZIF precursor at 300 °C in air for 3 h transforms it into rGO@ZnCo_2_O_4_ (GZC‐300) 2D nanosheets with the inherited morphology. This well‐preserved N‐doped bimetal oxide 2D nanocomposite with BET surface area of 892 m^2^ g^−1^ and high electrical conductivity proves to be excellent photocatalyst for NO oxidation with the conversion of 92.6% under sunlight.^[^ ^130^ ^]^

Semiconducting heterojunctions exhibit higher photocatalytic activities because the spatial distribution of photogenerated holes and electrons reduces the charge recombination, which results in improved charge separation and transfer, consequently enhanced photocatalytic performances. For example, 2D g‐C_3_N_4_/TiO_2_ nanosheets prepared by in situ calcination of the mixture of as‐prepared Ti‐MOF MIL‐125 (Ti) ground with melamine powder at 500 °C under air atmosphere. The 2D g‐C_3_N_4_/TiO_2_ nanosheets with TiO_2_ nanoparticles embedded in 8 wt% g‐C_3_N_4_ exhibit BET surface area of 177.3 m^2^ g^−1^ and enhanced photodegradation of MB under visible light. The valence and conduction band edges of the TiO_2_ nanoparticles and the g‐C_3_N_4_ nanosheets are positioned at such energies that they form direct Z‐scheme heterojunctions which spatially separate the oxidation sites on TiO_2_ and the reduction sites on g‐C_3_N_4_, suppressing the charge recombination.^[^ ^131^ ^]^ Recently, MOF derived multimetal compounds embedded in g‐C_3_N_4_ (such as CoFe_2_O_4_/Fe_2_O_3_/g‐C_3_N_4_) have also been reported as excellent photocatalysts with improved performance toward photodegradation of emerging pharmaceutical and organic pollutants.^[^ ^164^ ^]^ From the above mentioned few examples, it can be seen that it is challenging to prepare MOF derived 2D nanocomposites due to the difficulties to achieve the uniform growth of MOF with a controllable thickness on the 2D external templates. The rationally designed transition metal nodes and organic linkers can form a variety of simple 2D MOF precursors with a high density of uniformly distributed exposed catalytic active sites. Moreover, a variety of MOFs can be grown on 2D external templateds other than carbon to further explore the structures‐application relationships of the resulting 2D composites.

#### 4.2.4 3D Nanocomposites

##### Nanocubes

Almost a decade ago, in one of the early efforts, direct carbonization of as‐prepared cubic MOF‐5 precursor in an inert atmosphere at 600 °C resulted in a well‐defined cubic ZnO/C nanocomposite with BET surface area of 576 m^2^ g^−1^. This self‐templated MOF derived ZnO/C nanocomposite showed exceptionally high adsorption and photodegradation of RhB due to the *π*–*π* interactions between the MOF derived graphitic carbon layer with sp^2^ bonding and the aromatic rings of the organic dye molecules.^[^ ^62^ ^]^ Such attempt to pyrolyze MOFs offered the opportunities to understand these porous coordination polymers as sacrificial templates for the formation of 3D porous nanocomposites.^[^ ^62^ ^]^ Recently, our group has reported that the porous carbon matrix can be in situ functionalized with carboxylate functional groups (—COOH) in the presence of water vapor as a mild oxidizing agent during the direct carbonization of self‐templated MOF‐5. This simple one‐step pyrolysis of the as‐prepared cubic MOF precursor at 800 °C in water vapor produces C‐doped ZnO nanoparticles uniformly distributed in carboxylate group functionalized 3D porous carbon matrix. This cubic ZnO/C nanocomposite exhibits excellent adsorption and photodegradation of MB under visible light due to the high BET surface area of 350 m^2^ g^−1^ and an appropriate pore diameter of around 1.4 nm and narrow energy bandgaps.^[^ ^83^ ^]^ To understand the role of physicochemical properties and morphology on the photocatalytic performance, a systematic investigation on the ZnO/C composites derived from self‐templated polyhedral ZIF‐8, spherical MOF‐74 and cubic MOF‐5 precursors at 800 °C in water vapor saturated argon atmosphere has been carried out. The results show that the crystallinity of ZnO nanoparticles, the narrowed energy bandgap due to the doping of N and C species, the BET surface area and pore size distribution of the carbon matrix and the accessible active sites together with the surface functionalities play important role in photocatalytic degradation of MB under UV and/or visible light.^[^ ^152^ ^]^

##### Nanodiscs

Simple one‐step pyrolysis of self‐templated MOF, MIL‐125 precursor at high temperature in an inert atmosphere can result in the formation of highly stable 3D disc‐like TiO*_x_*/C composites.^[^ ^165^ ^]^ By controlling the pyrolysis temperature, the crystallinity and polymorphic phase ratios (anatase/rutile/brookite) of MOF derived TiO_2_ nanoparticles can be tailored. In MIL‐125 derived composite, TiO_2_ of anatase phase forms at pyrolysis temperatures below 600 °C, whereas rutile phase emerges at pyrolysis temperature above 600 °C. However, at 1000 °C, Ti_3_O_5_ forms, which is embedded in porous carbon matrix with BET surface area of 329 m^2^ g^−1^. The high surface area and conductive carbon matrix make this nanocomposite excellent photocatalyst for dye degradation.^[^ ^165^ ^]^ Recently, Lin et al. demonstrated that Co‐decorated MIL‐125 can be employed as a sacrificial self‐template to derive core/shell C–TiO_2_/CoTiO_3_ composites, forming a type‐II heterojunction, with an overall 3D disc‐like (or nanocake) morphology. The derived composites exhibited promising photocatalytic removal of (10 mg L^−1^) antibiotics ciprofloxacin (CIP) (99.6%) from aqueous solution under visible light for 120 min. The narrowing of energy bandgaps of C–TiO_2_ (2.94 eV) and CoTiO_3_ (2.29 eV) and formation of type‐II heterojunction extended the visible light absorption region, which lead to a higher photocatalytic degradation and removal of antibiotics under the visible light.^[^ ^166^ ^]^ To diversify the photocatalytic applications of the derived composites, further investigations are necessary to optimize the pyrolysis conditions to tailor the textural properties, polymorph structures, and formation of multimetallic compounds through the rationally selected functional MOF precursors.

##### External‐Templated 3D Nanocages and Nanosponges

The generation of 3D nanocomposites is not limited to self‐templated MOF precursor approach. The external‐templated MOF hybrid precursor method opens a door of opportunities to fabricate a large variety of 3D nanocomposites such as nanofoams, nanocages, nanosponges and graphitized carbon cloths.^[^ ^12^, ^39^, ^72^, ^167^ ^]^ Rationally designed external‐templated MOF hybrid precursors offer relatively easy ways to engineer the physicochemical properties of the derived nanocomposites. For example, ZIF‐8 can be grown on a porous 3D graphene network (3DGN) in methanolic solution at room temperature. Heat treatment of this external‐templated ZIF‐8/3DGN in argon followed by annealing in air below 400 °C can produce ZnO coated 3DGN composite (ZnO/3DGN).^[^ ^168^ ^]^ Benefiting from the 3D porous graphene network as a backbone, this nanocomposite shows good structural stability. The photocatalytic performance is improved due to the better charge conductivity of 3DGN as well as the high surface area of the ZnO/3DGN composites. The effective interfacial contact between the MOF derived ZnO nanoparticles and the 3DGN can minimize the recombination of the photoinduced holes and electrons during the photocatalytic process.

**Figure 11:**
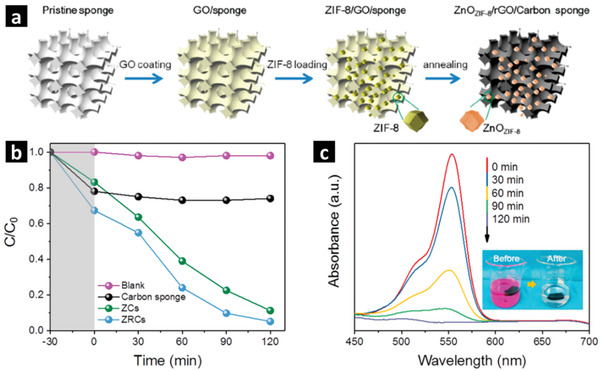
a) Schematic diagram of the formation process of ZnO_ZIF‐8_/rGO/carbon sponge. b) Photodegradation of RhB under UV–vis light irradiation. c) Change in intensity of absorption peak of RhB with the progression of time during photocatalysis (inset shows the change in color of RhB dye before and after complete photodegradation). Adapted with permission.^[^ ^167^ ^]^ Copyright 2018, American Chemical Society.

Usually, the MOF derived composites are in the form of powder, which in some cases do not conform to the practical requirements due to their poor dispersibility in certain solutions. The external‐templated MOF precursor approach can be advantageous to fabricate MOF derived nanocomposites with porous metal oxides uniformly loaded on the surfaces of rGO, which is coated on the carbon sponge frameworks. Such monolithic microreactor‐like 3D nanostructures offer higher structural stability and good opportunities in morphological control. For example, ZnO nanocages decorated on rGO coated 3D carbon sponge (ZnO/rGO/carbon sponge) has been obtained from direct calcination of dip‐coated ZIF‐8 on graphene oxide coated melamine foam at 350 °C. **Figure** **11** shows the schematic diagram of the fabrication process of ZnO_ZIF‐8_/rGO/Carbon sponge. This external‐templated N/C‐codoped ZnO decorated 3D nanosponge microreactor proved to be effective for both photodegradation of organic pollutants and photocatalytic H_2_ evolution under simulated sunlight. Such 3D nanocomposite can offer high structural stability as well as multiple functionalities as absorbent and photocatalyst.^[^ ^167^ ^]^

#### 4.2.5 Summary of MOF Derived Nanocomposites for Photodegradation of Organic Pollutants

MOF derived nanocomposites with diverse dimensions and a large variety of morphologies with in situ tunable properties can be derived from MOF precursors via either self‐templated or external‐templated methods. The morphology of the derived nanocomposites primarily depends upon the morphology of the MOF used as a precursor and/or sacrificial template. These derived composites offer great symbiotic relationships between different structural, textural, physicochemical and semiconducting properties for excellent photocatalytic applications. Some self‐templated and external‐templated MOF derived nanocomposites as photocatalysts for photodegradation of organic pollutants or dyes are summarized in **Table** **3**. As shown in Table 3, some transition metals such as Zn, Ti, Fe, Cu and Cd‐based‐MOF derived composites have been reported for photodegradation of organic dyes, industrial waste chemicals and pharmaceutical contaminants. Nevertheless, more detailed and in‐depth investigations need to be carried out to explore new types of MOF derived nanostructures such as multilayered core/shells, 2D single/multilayered optimized nanosheets and novel 3D morphologies that are from not only metal oxides but also other metal compounds such as metal sulfides and metal phosphides. Moreover, the formation of MOF derived complex nanostructures needs to be further investigated by controlling the molar/atomic ratios of the (multi)metal compositions and modifying the in situ formed porous carbons to fully understand and enhance the structural stability as well as their photodegradation efficiencies.

**Table 3:** Comparison of selected MOF derived nanocomposites for photodegradation of organic pollutants

				Experimental conditions			
MOF precursor	Catalyst	Morphology	Pollutant	Amount and concentration of pollutant	Light source and exposure time	Degradation efficiency [%]	Refs.
MIL‐53(Fe)	*γ*‐Fe_2_O_3_/C	Polyhedral	MG with H_2_O_2_	5 mL (500 mg L^−1^)	Sunlight 3 h	100	^[^ ^155^ ^]^
ZIF‐8	ZnO/N–C	Rhombic dodecahedron	MB	100 mL (20 mg L^−1^)	>420 nm 200 W 4 h	99	^[^ ^84^ ^]^
ZIF‐8	C–N–ZnO	Rhombic dodecahedron	MB	1000 mL (10 mg L^−1^)	Simulated solar light 2, 5 h	100	^[^ ^75^ ^]^
ZIF‐8/CdS	CdS/MPC	Polyhedron	Antibiotics CPX	100 mL (20 mg L^−1^)	>420 nm 300 W Xe lamp 1.5 h	90.5	^[^ ^150^ ^]^
MOF‐5	C@ZnO	Octahedral	RhB	100 mL	>420 nm 350 W Xe lamp 3.5 h	≈90	^[^ ^156^ ^]^
HKUST‐1‐P	P‐C/Cu_2_O	Octahedral	Phenol	10 mL (40 mg L^−1^)	>420 nm 350 W Xe lamp 1.5 h	99.8	^[^ ^151^ ^]^
MOF‐5	ZnO@C	Cubic	RhB	24 mg L^−1^	UV light 12 h	98	^[^ ^62^ ^]^
Fe_3_O_4_@HKUST‐1	Fe_3_O_4_@C/Cu	Core/shell	MB with H_2_O_2_	100 mL (20 mg L^−1^)	>420 nm 500 W Xe lamp 2.5 h	100	^[^ ^93^ ^]^
Zn/Co–ZIF	ZnO@C–N–Co	Core/shell	MO	50 mL (16.5 mg L^−1^)	UV light 300 W Xe lamp 2.5 h	99.5	^[^ ^111^ ^]^
ZIF‐8/rGO	ZnO/rGO	Core/shell	Orange II	40 mL (10 mg L^−1^)	Simulated solar light 5 h	100	^[^ ^113^ ^]^
ZIF‐67/GO	Co–G	Nanosheet	AY and PMS	500 mL (100 mg L^−1^)	UV light 2 h	≈80	^[^ ^169^ ^]^
Cd‐MOF	CdS/N–C	Nanorod	Antibiotics TC	100 mL (40 mg L^−1^)	>420 nm 300 W Xe lamp 1 h	83	^[^ ^158^ ^]^
MIL‐88A	Fe_2_O_3_/C	Nanorod	RhB with peroxide and persulfate	500 mL (10 mg L^−1^)	UV light 1.5 h	≈90	^[^ ^112^ ^]^
MIL‐88‐Fe	TiO_2_@C/FeTiO_3_	Nanotube	Phenol	(5 mg L^−1^)	Simulated solar light 500 W Xe lamp 3 h	80	^[^ ^159^ ^]^
MOF‐5	ZnO/C	Cubic	MB	50 mL (20 mg L^−1^)	>420 nm 200 W 3 h	99	^[^ ^83^ ^]^
ZnCo–ZIF	rGO@ZnCo_2_O_4_	Nanosheet	NO oxidization	Gas phase 10.8 L continuous flow	Solar light 300 W halogen lamp 1 h	92.6	^[^ ^130^ ^]^
ZIF‐8/GO/melamine foam	ZnO/rGO/C	3D sponge	RhB	100 mL (10 mg L^−1^)	Simulated solar light 300 W Xe lamp 2 h	99	^[^ ^167^ ^]^
Cu‐MOF	N–Cu_2_O/C	2D nanoplatelets	MO	50 mL (50 mg L^−1^)	>430 nm 300 W Xe lamp 3 h	90	^[^ ^163^ ^]^

### 4.3 MOF Derived Nanocomposites for Photocatalytic CO_2_ Reduction

Rationally designed MOF derived nanocomposites with suitable properties can be promising candidates for highly efficient CO_2_ adsorption and photocatalytic reduction into hydrocarbons. High performance nanomaterials for sunlight‐driven CO_2_ reduction should possess high BET surface area, appropriate pore structure and accessible active sites for enhanced CO_2_ adsorption and chemical conversion. The challenge is that CO_2_ molecule is thermodynamically stable with closed‐shell configuration and without dipole moment. Therefore, relatively high activation energy and appropriate catalytic materials with finely tuned properties are required to realize the endothermic reactions that can readily reduce and convert CO_2_ into useful hydrocarbons. Thereby, suitable nanomaterials with narrow energy bandgaps and optimized band edge positions are necessary to maximize the light‐harvesting and charge separation/transfer, consequently, to achieve significant enhancement of the photocatalytic CO_2_ conversion rate and selectivity.^[^ ^27^, ^34^, ^90^ ^]^

#### 4.3.1 Core–Shell Structures

**Figure 12:**
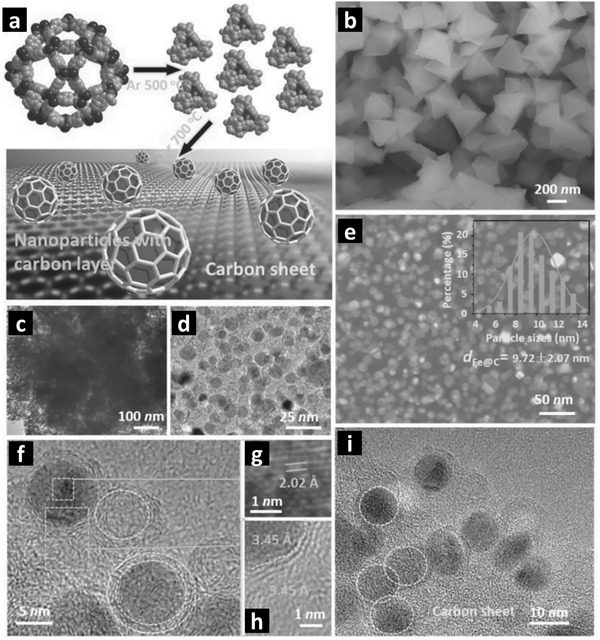
a) Schematic representation of the self‐templated MIL‐101(Fe) derived core/shell Fe@C nanocomposite. b) SEM image of crystals of the sacrificial template MIL‐101. c,d) Low‐resolution TEM. e) STEM images of the Fe@C nanocomposite where inset in (e) shows the particle size distribution of the Fe nanoparticles encapsulated in carbon shell. f–i) High‐resolution TEM images of the core/shell Fe@C nanocomposite; the carbon shells and encapsulated metal nanoparticles can be seen. g) (1 1 0) lattice spacing of Fe and h) interplane (0 0 2) space of graphene, respectively. Reproduced with permission.^[^ ^94^ ^]^ Copyright 2016, Wiley‐VCH.

It is well understood that catalytically active metal particles supported on graphitic carbon exhibit excellent performance for CO_2_ reduction and conversion to hydrocarbons. Several computational and experimental studies on metal/carbon (M/C) composites for CO_2_ conversion application have suggested that the improvement in their photocatalytic performance as compared to the bare metal nanoparticles is due to the modified electronic structure and work function caused by the presence of carbon species.^[^ ^170^, ^171^, ^172^ ^]^ Self‐templated MOF precursors are excellent candidates for simple in situ fabrication of M/C, MO*_x_*/C or M‐MO*_x_*/C composites. Zhang et al. fabricated Fe@C core–shell nanocomposites by a controlled two‐step thermal treatment of self‐templated MOF, MIL‐101(Fe) at 500 and 700 °C in argon. As shown in **Figure** **12**, the decomposition of MIL‐101 at 500 °C under argon formed predominantly Fe_3_C particles along with a trace amount of Fe_3_O_4_ nanoparticles. Raising the carbonization temperature to 700 °C resulted in the reduction of Fe_3_O_4_ and Fe_3_C into the final Fe@C nanocomposite without agglomeration. The presence of Fe nanoparticles facilitated the graphitization of carbon that was derived from the organic linker, which resulted in a nanocomposite containing uniformly dispersed Fe nanoparticles of average diameter less than 10 nm coated with ultrathin graphitic carbon (1–3 layers). This core–shell nanostructure exhibited photocatalytic CO_2_ conversion activity of 18.3 mmol g_cat_^−1^ h^−1^ into CO with high selectivity of 99.9%. The experimental results were supported by the density functional theory (DFT) simulation, which showed that the improved CO_2_ conversion under sunlight in broader wavelength range could be attributed to the improved light absorption by the ultrathin carbon layer and the catalytic activity of the Fe core. The absorbed UV light by the Fe nanoparticles produced localized surface plasmon resonance and activated the nonpolar CO_2_ molecules. Moreover, the visible/IR part of incident light absorbed by the catalyst simultaneously caused a thermal effect that accelerated the CO_2_ conversion reaction. The plasmon–photon coupling at the surface of the Fe core enhanced the electron generation leading to more efficient CO_2_ activation. In addition, the ultrathin few layers of porous carbon shell assisted the easy desorption of produced CO from the surface of the catalyst Fe.^[^ ^94^ ^]^

**Figure 13:**
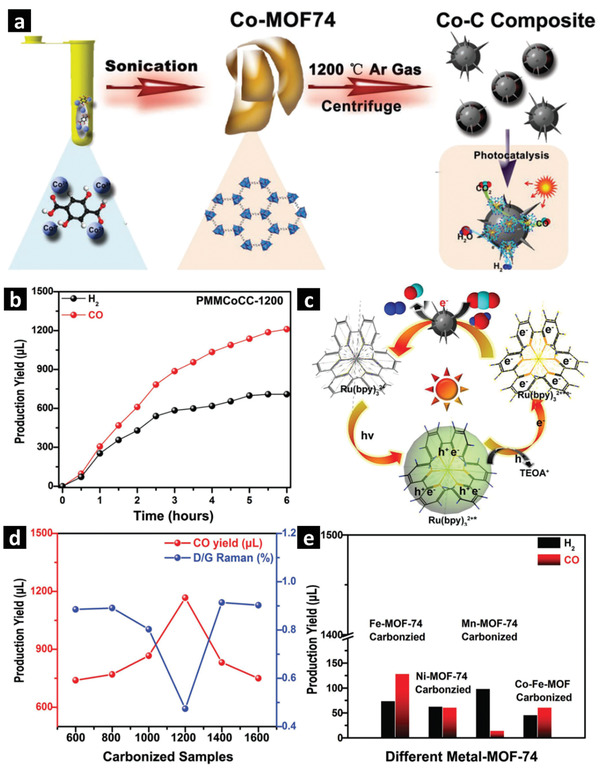
a) Schematic diagram for the synthetic strategy of Co‐MOF‐74 derived Co–C nanocomposite (PMMCoCC‐1200). b) Photocatalytic CO (red line) and H_2_ (black line) evolution under the visible light. c) Proposed mechanism of the photocatalytic CO_2_ reduction to CO by Co–C nanocomposite (PMMCoCC‐1200) in the presence of electron donor TEOA and photosensitizer Ru(bpy)_3_^2+^. d) The relationship between the degree of graphitization (*I*_D_/*I*_G_ ratio characterized by Raman spectra) and the CO_2_ reduction by the samples derived at different carbonization temperatures. e) Comparison of CO_2_ reduction by different metal/carbon composites derived from M‐MOF‐74 (M = Fe, Ni, Mn, and mixed Co–Fe) under the identical carbonization conditions (1200 °C under an inert atmosphere). Adapted with permission.^[^ ^109^ ^]^ Copyright 2018, Wiley‐VCH.

Other transition metal‐based MOF precursors can be used to derive metal/porous carbon nanocomposites with different metal/carbon ratios and tunable porosities. For example, M‐MOF‐74 precursor (M = Co, Ni, Fe, Mn) has been carbonized at a higher temperature to obtain magnetic metal nanoparticles embedded in highly graphitized porous carbon.^[^ ^109^ ^]^ Zhao et al. reported a highly graphitized porous metallic and magnetic Co–C composite (PMMCoCC‐1200) derived from self‐templated Co‐MOF‐74 at 1200 °C with a core–shell morphology. In the presence of photosensitizer Ru(bpy)_3_^2+^ and sacrificial electron donor TEOA, the PMMCoCC‐1200 showed good CO_2_ reduction activity to CO compared with other MOF‐74 derived M/C composites (M = Fe, Ni, Mn). The better photocatalytic performance of Ni‐MOF‐74 derived PMMCoCC‐1200 composite under visible light (>420 nm) can be attributed to the high electric charge conductivity of the graphene‐like carbon and the well‐exposed metal cobalt active sites. The better CO_2_ adsorption capacity of the porous carbon matrix also offers a promising opportunity of CO_2_ molecules proximity to the metal sites. Moreover, the degree of graphitization, the uniform morphology (**Figure** **13**) and the M/C ratio can be optimized by adjusting the carbonization conditions. Besides, the magnetic property of the M/C nanocomposites offers an advantage of easy collection and good recyclability of the photocatalyst after the heterogeneous photocatalytic reaction.^[^ ^109^ ^]^

#### 4.3.2 Nanosheets

MOF derived metal oxides or metal sulfides with appropriate semiconducting properties and suitable energy band positions can be utilized for photocatalytic CO_2_ reduction. Recently, Cu‐MOF, HKUST‐1 derived C–Cu_2−_ *_x_* S tubular‐shaped nanostructures combined with g‐C_3_N_4_ nanosheets derived from polymerization of urea in argon have been reported.^[^ ^108^ ^]^ These optimized ternary C–Cu_2−_ *_x_* S/g‐C_3_N_4_ heterojunction nanosheets containing 0.71 wt% of C–Cu_2−_ *_x_* S showed photocatalytic CO_2_ reduction activity of 1.06 mmol g_cat_^−1^ h^−1^ with CO selectivity of 97% in the presence of water vapor. Upon visible light irradiation, most of the photons were absorbed by Cu_2−_ *_x_* S generated electron–hole pairs. Due to the formation of heterojunctions at the interface of the nanosheets, the photogenerated electrons migrated to the MOF derived carbon matrix and triggered the CO_2_ reduction reaction assisted by the proton (H^+^) originated from H_2_O vapor whereas the photoinduced holes on g‐C_3_N_4_ oxidized water to generate O_2_. Compared to the bare g‐C_3_N_4_, this sevenfold enhancement in photocatalytic activity of CO_2_ reduction can be attributed to the better visible light absorption by Cu_2−_ *_x_* S, efficient charge separation and suppressed charge recombination by the heterojunctions. Despite the great possibilities in the synthesis of a large variety of MOF derived nanocomposites for photocatalytic CO_2_ reduction, to date, only a few studies are available. Therefore, more research efforts are expected to be devoted to this exciting area to synthesize rationally designed self‐templated or external‐templated MOF precursors. Nanocomposites can be derived from those MOF precursors to investigate the relationships between the structures, physical and chemical properties, and the photocatalytic performance, dedicating to enhance the solar‐light‐driven CO_2_ conversion efficiencies and selectivity.^[^ ^108^ ^]^

## 5 Summary and Perspective

MOF precursors/templates, synthesized with countless combinations of metal ions/clusters and organic linkers, can derive a large variety of novel nanocomposites including pure metals, multimetals, alloys, oxides, carbides, chalcogenides and phosphides embedded in the porous carbon matrix. These MOF derived nanocomposites, in general, show better photocatalytic performance compared to the pure metal compounds due to the presence of conductive carbon matrix, which offers enhanced adsorption/absorption capacities, well‐exposed active sites, and improved charge separation/migration pathways. Moreover, in MOF derived nanocomposites, the structures and chemical compositions play significant roles in the photocatalytic performance. Though a variety of structures with diverse morphologies, controllable textural properties and modifiable chemical compositions have been reported, it is still a great challenge to achieve precise control at the atomic scale to customize the nanocomposites with application‐specific properties for maximum performance. There is, therefore, a vast scope to prepare rationally designed novel MOF precursors and sacrificial templates apart from the commonly known ones such as ZIFs, MOFs, UiOs, and MILs, to derive energy bandgap finely tunable and highly desirable nanocomposites with optimized physicochemical properties for photocatalysis applications.

The approach of pyrolysis of external‐templated MOF precursors to obtain nanocomposites offers a diverse choice of MOFs and the external templates (morphology modulators), but it involves multistep synthetic procedures making this method costly and commercially unfavorable. The method of heat‐treatment of self‐templated MOF precursors under different gas atmospheres to generate nanocomposites is a simple and low‐cost direct one‐step method. However, this approach suffers from limited control over the morphologies and textural properties of the targeted nanocomposites. Therefore, new synthesis methods and pyrolysis strategies are needed for precise control over molar and atomic ratios of MOF precursors (components, reactants and solvents), as well as the role of pyrolysis conditions (gaseous atmosphere, pyrolysis temperature, heating rate and dwell time of heating), require further detailed investigations. New combinations of multimetallic guest species (in particular transition metals) encapsulated in MOF precursors are also needed to be rationally designed to derive complex, multilevel hierarchical nanostructures that can improve structural stabilities and generate synergistic effects to enhance the photocatalytic performance. Compared to other synthesis approaches, MOF derived composites enable the uniform distribution of metal components/particles with negligible agglomerations in the carbon matrix. In situ modification of the particle sizes by well‐regulated pyrolysis conditions, energy bandgap engineering by the incorporation of metal and nonmetal dopants (C, N, S, P, etc.) as well as the formation of optimized semiconducting heterojunctions to tune the energy band positions need to be further explored for enhanced visible light absorption, photoinduced electric charge generation and separation with suppressed charge recombination.

The degree of graphitization with adjustable *I*_D_/*I*_G_ ratio, chemical compositions and textural properties of the MOF derived carbon matrix also plays a crucial role in determining the photocatalytic activity of the MOF derived nanocomposites. Systematic operando/in situ investigations by advanced spectroscopic techniques are required to help understand the transition of the precursors to the nanocomposites, surface‐functionalities and the formation of defects. These properties are important in the absorption of the incident light, electrical charge conductivity and reduction–oxidation reactions at photocatalytic active sites. Such in‐depth studies may reveal interesting new features to further expand our understanding of the structure–property–application relationships, which will enable us to rationally design and develop high‐performance novel photocatalysts without relying on expensive noble metals.

Compared with photocatalytic H_2_ evolution and photocatalytic degradation of organic pollutants, only a few studies are available on MOF derived nanocomposites for photocatalytic CO_2_ reduction and conversion into value‐added hydrocarbons. It is a less explored research area, therefore more effort needs to be devoted to this highly interesting and important field. A variety of novel MOF derived nanocomposites are expected to be synthesized for highly efficient photocatalytic CO_2_ reduction because the combination of metal compounds and porous carbon matrix can be advantageous to simultaneously capture and reduce CO_2_ into different value‐added products such as CO, CH_4_, CH_3_OH and C_2_H_5_OH. In addition, more detailed investigations by in situ ultrafast optical spectroscopic techniques are needed to in‐depth understand the photocatalytic CO_2_ reduction mechanisms including light absorption, charge generation and separation, charge migration and redox reactions in the MOF derived nanocomposites.

Apart from the experimental work, theoretical studies are also essential to understand the role of each parameter (metallic species, heterojunctions, dopants, defects, functionalities, pore shape/size, carbon matrix, energy band structures and active sites) and relevant properties in sunlight‐driven photocatalytical applications. In particular, emerging tools such as computational and machine learning algorithms can significantly help in rational design, classification, clustering and dimensionality reduction tasks of high‐dimensional, heterogeneous data.

MOF derived composites are commonly obtained in form of powders. However, for practical applications of such photocatalysts, other forms of the materials may be favorable. For instance, MOF derived composites can be 3D printed in desirable shape/size and installed as “plug‐and‐play” type devices. Once succeed, it will remarkably enhance the ease of handling such materials at a very large scale. Moreover, a few MOFs have already been commercialized at affordable prices and mass production can further decrease their prices. New cost‐effective and simple synthesis methods can make MOF derived photocatalysts an important alternative to the conventional energy materials. With more studies on novel MOF derived nanocomposites and the in‐depth exploration of the photocatalytic reaction mechanisms, these MOF derived nanocomposites are expected to be promising photocatalytic materials for the production of clean energy and the mitigation of environmental issues in the near future.

## Conflict of Interest

The authors declare no conflict of interest.
